# Implementation of Web-Based Psychosocial Interventions for Adults With Acquired Brain Injury and Their Caregivers: Systematic Review

**DOI:** 10.2196/38100

**Published:** 2022-07-26

**Authors:** Melissa Miao, Rachael Rietdijk, Melissa Brunner, Deborah Debono, Leanne Togher, Emma Power

**Affiliations:** 1 University of Technology Sydney Sydney Australia; 2 University of Sydney Sydney Australia

**Keywords:** complexity, implementation science, internet interventions, acquired brain injury, delivery of health care, caregivers, digital health, psychosocial interventions, psychosocial, brain injury, mobile phone

## Abstract

**Background:**

More than 135 million people worldwide live with acquired brain injury (ABI) and its many psychosocial sequelae. This growing global burden necessitates scalable rehabilitation services. Despite demonstrated potential to increase the accessibility and scalability of psychosocial supports, digital health interventions are challenging to implement and sustain. The Nonadoption, Abandonment, Scale-Up, Spread, and Sustainability (NASSS) framework can offer developers and researchers a comprehensive overview of considerations to implement, scale, and sustain digital health interventions.

**Objective:**

This systematic review identified published, peer-reviewed primary evidence of implementation outcomes, strategies, and factors for web-based psychosocial interventions targeting either adults with ABI or their formal or informal caregivers; evaluated and summarized this evidence; synthesized qualitative and quantitative implementation data according to the NASSS framework; and provided recommendations for future implementation. Results were compared with 3 hypotheses which state that *complexity* (dynamic, unpredictable, and poorly characterized factors) in most or all NASSS domains increases likelihood of implementation failure; success is achievable, but difficult with many *complicated* domains (containing multiple interacting factors); and *simplicity* (straightforward, predictable, and few factors) in most or all domains increases the likelihood of success.

**Methods:**

From a comprehensive search of MEDLINE, EMBASE, PsycINFO, CINAHL, Scopus, speechBITE, and neuroBITE, we reviewed primary implementation evidence from January 2008 to June 2020. For web-based psychosocial interventions delivered via standard desktop computer, mobile phone, tablet, television, and virtual reality devices to adults with ABI or their formal or informal caregivers, we extracted intervention characteristics, stakeholder involvement, implementation scope and outcomes, study design and quality, and implementation data. Implementation data were both narratively synthesized and descriptively quantified across all 7 domains (condition, technology, value proposition, adopters, organization, wider system, and their interaction over time) and all subdomains of the NASSS framework. Study quality and risk of bias were assessed using the 2018 Mixed Methods Appraisal Tool.

**Results:**

We identified 60 peer-reviewed studies from 12 countries, including 5723 adults with ABI, 1920 carers, and 50 health care staff. The findings aligned with all 3 hypotheses.

**Conclusions:**

Although studies were of low methodological quality and insufficient number to statistically test relationships, the results appeared consistent with recommendations to reduce complexity as much as possible to facilitate implementation. Although studies excluded individuals with a range of comorbidities and sociocultural challenges, such simplification of NASSS domain 1 may have been necessary to advance intervention value propositions (domain 3). However, to create equitable digital health solutions that can be successfully implemented in real-world settings, it is recommended that developers involve people with ABI, their close others, and health care staff in addressing complexities in domains 2 to 7 from the earliest intervention design stages.

**Trial Registration:**

PROSPERO International Prospective Register of Systematic Reviews CRD42020186387; https://www.crd.york.ac.uk/prospero/display_record.php?ID=CRD42020186387

**International Registered Report Identifier (IRRID):**

RR2-10.1177/20552076211035988

## Introduction

### Background

More than 135 million people worldwide live with acquired brain injuries (ABIs), such as stroke and traumatic brain injury (TBI) [1]. The number of people with ABI is projected to grow [2], increasing global need for rehabilitation services [1,2], including supports to manage the complex and ongoing psychosocial impact of ABI on relationships [3,4], mental health [5,6], and employment [7,8]. For these rehabilitation services to be provided at scale, they must be effectively integrated into health care systems [1].

Longstanding challenges in the implementation of evidence-based care have led to the emergence of implementation science research [9]. This includes a specific focus on digital health implementation [10,11]. Despite demonstrated potential to increase the accessibility and scalability of psychosocial supports [12,13], digital health interventions are challenging to implement and sustain [11,14,15]. Current evidence indicates that digital health implementation challenges are predominantly organizational, systemic, and sociotechnical in nature, including interrelated challenges of resources, workflows, interoperability, and legislation [10,15,16]. Therefore, understanding and addressing these challenges require a comprehensive, complexity-based approach, in which the complex, adaptive nature of health care systems, actors, and technologies, as well as the interactions between them, are recognized [17-19].

From this complexity paradigm [18-20], the Nonadoption, Abandonment, Scale-up, Spread, and Sustainability (NASSS) framework [17] offers developers, practitioners, and researchers a comprehensive synthesis of considerations to implement, scale, and sustain digital health interventions, to ensure that critically important systemic and organizational factors are not overlooked. The NASSS framework includes 7 domains of digital health implementation: condition, technology, value proposition, adopters, organization, wider system, and their interaction over time [17]. Each domain includes multiple subdomains, with published definitions of how each specific subdomain can be made *simple*, *complicated*, or *complex* [17]. A complexity paradigm has not yet been adopted for digital interventions targeting ABI, despite both the prevalence of this condition [1] and the value of a condition-specific focus from both theoretical [20] and stakeholder perspectives [21].

To date, the NASSS framework has been used to narratively synthesize digital health implementation findings from informal care [22], mixed home care [23], and video consultations [24] in various populations. However, it has not yet been used to underpin deductive extraction and analysis of qualitative data [25] or quantitative analyses in relation to current hypotheses concerning the potential role of complexity in implementation success [20]. Digital health implementation reviews to date have also relied on generic implementation frameworks [26-28], despite their poor fit to digital health [29]. There is therefore a need to examine existing implementation evidence specific to digital health, ABI, and its psychosocial sequelae, and to do so using a comprehensive framework that acknowledges the complexity of implementing, scaling, and sustaining digital health interventions in real-world settings, if we are to enable these interventions to succeed at a scale that can reach and support current and future global needs.

### Aims

Based on a previously published protocol [30], the aims of this review were as follows:

1. Identify, evaluate, and summarize the strength and nature of implementation evidence for web-based psychosocial interventions targeting either people with ABI or their caregivers or both.

2. Synthesize qualitative and quantitative implementation data according to the NASSS framework.

3. Provide recommendations for future implementation based on this synthesis.

A subsequently introduced aim was as follows:

4. Compare findings with 3 hypotheses concerning the NASSS framework [20], which state:

- *Hypothesis 1: “If most or all of the domains can be classified as simple, an intervention is likely to be easy to implement and to be achieved on time and within budget”*;
- *Hypothesis 2: “If many domains are classified as complicated, the intervention will be achievable but difficult, and likely to exceed its timescale and budget”;*
- *Hypothesis 3: “If multiple domains are complex, the chances of the intervention succeeding at all are limited.”*

## Methods

### Review Registration and Protocol

This systematic review was prospectively registered in PROSPERO (International Prospective Register of Systematic Reviews; CRD42020186387) [31]. A published protocol [30], including the search strategy and selection criteria, was developed a priori. Subsequent protocol adjustments, with rationales, are reported in accordance with PRISMA (Preferred Reporting Items for Systematic Reviews and Meta-Analyses) 2020 guidelines [32].

### Search Strategy and Selection Criteria

**Figure 1:**
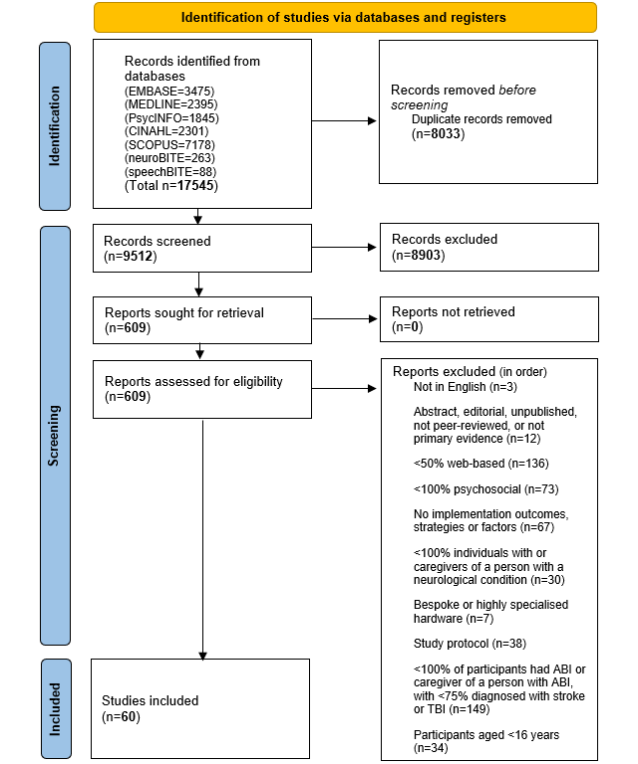
PRISMA (Preferred Reporting Items for Systematic Reviews and Meta-Analyses) 2020 flow diagram. ABI: acquired brain injury; TBI: traumatic brain injury.

A comprehensive search of 7 databases (MEDLINE, EMBASE, PsycINFO, CINAHL, Scopus, speechBITE, and neuroBITE) was conducted in mid-June 2020 as per the published protocol [30]. The original Population, Intervention, Comparison, Outcome, Study design–based search encompassed multiple neurological conditions (search strategy is available in Multimedia Appendix 1) and returned 17,545 results (refer to Figure 1 for PRISMA flow diagram). After removing duplicates, a total of 9512 titles and abstracts were independently screened using Covidence (Veritas Health Innovation) software [33] by 2 authors (MM and either MB, RR, EP, or DD), applying exclusion criteria in hierarchical order (as listed in Figure 1). There was 96.4% (9170/9512) agreement at the title and abstract level (ie, 3.6% disagreement, or 342/9512 conflicts, including agreed exclusions for conflicting reasons). Conflicts were resolved through consensus discussion by at least 3 authors. A total of 609 records were screened at the full-text level. Due to the high yield of full texts, a pragmatic protocol adjustment was required. Therefore, all full texts were screened by the first author (MM), and a second author (RR, EP, DD, or MB) independently screened 25.1% (153/609) rather than 100% of full texts. There was 82.4% (126/153) agreement in this quarter of full texts (ie, 17.6% disagreement, or 27/153 conflicts, including agreed exclusions for conflicting reasons). The conflicts were discussed by at least 3 authors and resolved by consensus. The team agreed that the reliability of the screening process was adequate for the first author to proceed independently.

Given the high yield of full texts, additional records were not sought as originally planned in the protocol [30]. Instead, additional criteria were selected to increase the review’s clinical relevance to our own implementation of web-based psychosocial interventions delivered via standard desktop computers and smart devices to adults with ABI and their communication partners [34]. These narrowed exclusion criteria were introduced in the following hierarchical order (Figure 1):

1. Less than 50% of the web-based intervention was delivered remotely; that is, web-based interventions accessed in a laboratory or clinic were excluded. For example, although Connor et al [34] examined a web-based brain training game, the study was excluded because it focused on in-person delivery on-site, accompanied by face-to-face treatment by a speech-language pathologist.
2. Less than 100% of the intervention was psychosocial in nature, that is, providing cognitive, behavioral, educational, communicational, or supportive care to both the person with the condition or their caregivers. Therefore, interventions with physical rehabilitation (eg, exercise programs, or physical therapy) or health informatics (eg, symptom monitoring, interprofessional communication, or care planning) components were excluded.
3. Less than 100% of participants were diagnosed with a neurological condition or the caregiver of such a person or the results of participants meeting this criterion could not be extracted.
4. The intervention required bespoke or highly specialized hardware beyond standard desktop computer, television, mobile phone, tablet, or virtual reality devices.
5. The record was a study protocol.
6. Less than 100% of intervention recipients were people with ABI or their caregivers, with <75% of the population having had a stroke or TBI or the caregiver of someone with these conditions.
7. Participants were aged <16 years.

The refinement in focus from neurological conditions in criterion 3 to the condition of ABI in criterion 6 aimed to reduce complexity in the first domain of the NASSS framework [20] by “scaling back on the kinds of illness or condition for which the technology is claimed to be useful.” It was also introduced to reflect stakeholders’ prioritization of the condition of ABI compared with other NASSS domains [21].

### Extraction

In total, 60 records were included for extraction against the NASSS framework [17]. Due to the high yield requiring in-depth application of the NASSS framework, a pragmatic deviation was required. Therefore, a second author (RR, MB, or DD) checked 25% (15/60) of extractions by the first author (MM) rather than independently extracting all full texts. Additional details were added or changes made in 1.18% (26/2205) of fields, confirmed via written consensus between the 2 rating authors and a third rater if necessary. To ensure consistency, the first author (MM) used a standardized extraction form (Multimedia Appendix 2 [17,36-38]), which included embedded logic via REDCap (Research Electronic Data Capture; the REDCap Consortium) [39], and the published definitions of (1) *complex*, *complicated*, or *simple* for each subdomain of the NASSS framework [17]; (2) each implementation outcome [36]; and (3) each question in the critical appraisal tool [37]. Text was extracted verbatim, with minimal paraphrasing as required for context.

The data extraction form drafted in our study protocol [30] was updated (Multimedia Appendix 2) to reflect the refined exclusion criteria. New extraction items were also added from a published taxonomy of digital health intervention features [38] to both consistently capture the diversity of interventions and technologies and incorporate implementation considerations identified by stakeholders in a concurrent study [21]. These included the order in which intervention contents were presented and the potential benefit of peer interaction. As described in our protocol [30], study quality, including sampling and nonresponse bias as applicable, was assessed across various study designs using the Mixed Methods Appraisal Tool (MMAT) [37]. Extraction was completed successively via REDCap, ensuring blinding to any emerging patterns until data from all 60 records had been extracted.

### Analysis

The high yield of this review enabled the quantification of complexity using descriptive statistics. The numbers of *complex*, *complicated*, and *simple* subdomains and domains and those containing *no information* were each subtotaled. Subdomains were classified according to their published definitions [17] as part of the extraction process (Multimedia Appendix 2). As no domain can be simpler than its constituent parts, each domain for each record was operationally classified according to the most complex subdomain present within that domain (Table 1). Implementation *success* or *failure* for each study was defined as whether the authors succeeded in achieving their specific implementation aims. *Can’t tell* was selected (Multimedia Appendix 2) for ambiguous implementation outcomes, such as inconclusive or insufficiently reported implementation results or conflicting implementation and effectiveness results. Such records were excluded, resulting in 75% (45/60) of the records being included in the descriptive analysis of complexity.

In accordance with our protocol [30], all quantitative results were analyzed in REDCap and Microsoft Excel (Microsoft Corporation) using descriptive statistics, and all qualitative results were narratively synthesized according to the NASSS framework [17].

**Table 1:** Operational classification of domain complexity according to subdomain complexity.

Domain complexity	General definition [17]	Subdomain complexity
Complex	“Dynamic, unpredictable, not easily disaggregated into constituent components”	Complex only; complex and complicated; or complex, complicated, and simple
Complicated	“Multiple interacting components or issues”	Complicated only or complicated and simple
Simple	“Straightforward, predictable, few components”	Simple only

## Results

### Note Regarding Style

Due to the high number of citations per descriptor, only essential in-text citations are provided. An appended Microsoft Excel spreadsheet containing all bibliographic and extracted data is provided as Multimedia Appendix 3.

### Implementation Evidence

**Figure 2:**
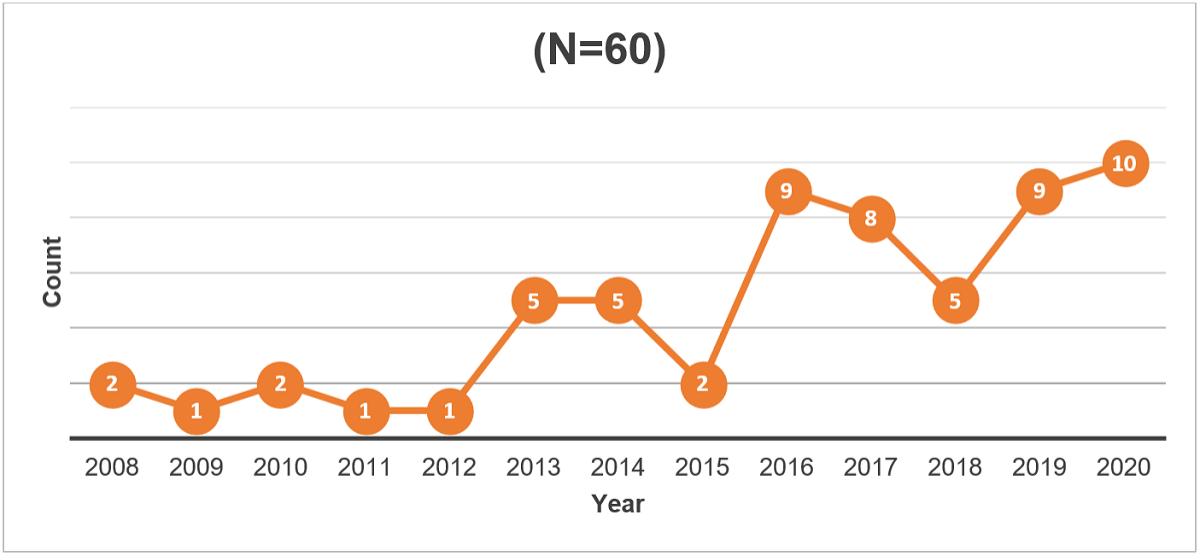
Publication year of included studies.

**Figure 3:**
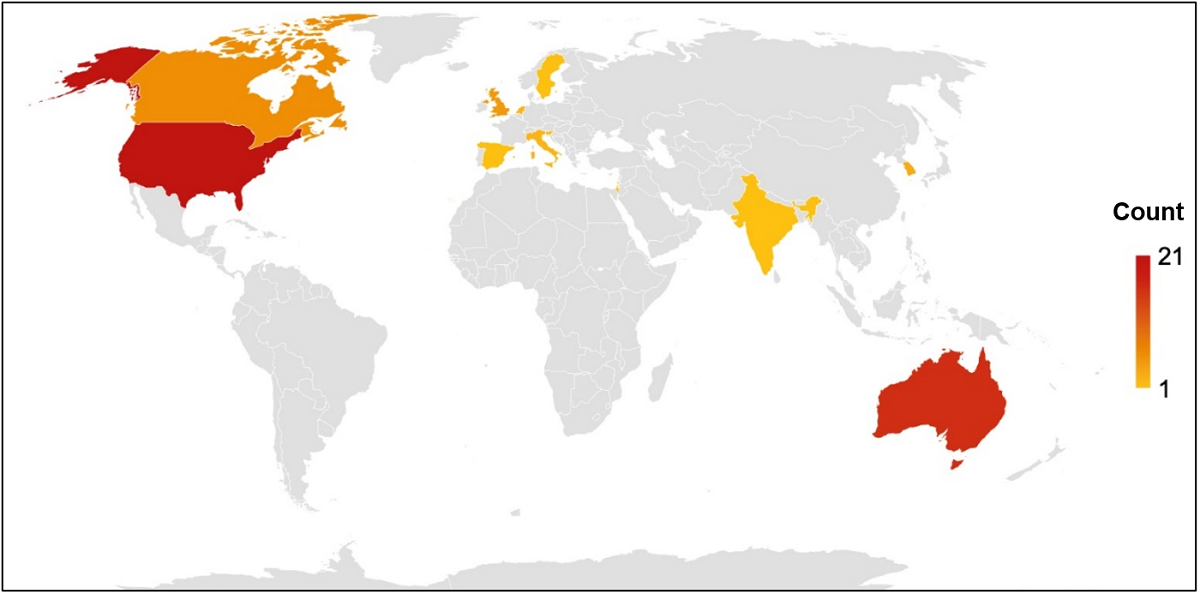
Country of origin of included studies.

More than two-thirds (41/60, 68%) of the reviewed studies were published in 2016 or later (Figure 2). Studies originated from 12 countries (Figure 3), with 3% (2/60) involving international collaboration [40,41]. The most studies were conducted in the United States (21/60, 35%), Australia (16/60, 27%), and Canada (6/60, 10%).

Overall, 13% (8/60) of studies used an implementation framework or theory. A generic framework was used in less than half (3/8, 38%) of these studies; Pitt et al [42,43] reported that their intervention had been developed according to a guide for complex intervention development that was not specific to digital health, while Rietdijk et al [44] referred to a generic framework for feasibility to inform study design. Almost twice as many (5/8, 63%) referred to a framework specific to the development of digital interventions, including web-based education [45], web-based programs [46], and user-centered design [47-49].

The most frequently used study design was mixed methods (22/60, 37%), followed by quantitative descriptive (17/60, 28%), quantitative nonrandomized trials (11/60, 18%), quantitative randomized controlled trials (RCTs; 8/60, 13%), and qualitative research (2/60, 3%). The MMAT definition for mixed methods inherently requires mixed methods studies to meet all 5 quality criteria. However, given the reviewed studies that used both qualitative and quantitative methods were typically of poor quality (refer to *Study Quality* section), a deviation was made in which the quality criteria for *mixed methods* were used to describe how these studies fell short of the definition, rather than immediately classifying all these studies as *Other* (Multimedia Appendix 2).

Among hybrid effectiveness implementation studies [50], Type 2 hybrids were the most common (22/60, 37%), followed by Type 1 (17/60, 28%), and Type 3 (4/60, 7%) [51-54]. Of the 60 studies reviewed, 3 (5%) were qualitative studies of implementation [42,55,56], and 14 (23%) used other nonhybrid designs. According to definitions by Proctor et al [36], the most frequently collected implementation measure was feasibility (37/60, 62%), followed by adherence or fidelity (27/60, 45%), satisfaction (22/60, 37%), usability (19/60, 32%), and acceptability (17/60, 28%). Appropriateness (9/60, 15%), cost-effectiveness (2/60, 3%), and other measures (5/60, 8%) were less frequently observed.

In terms of implementation outcomes, most studies (37/60, 62%) achieved the investigators’ specific implementation aims, while a minority (8/60, 13%) failed to do so (Multimedia Appendix 3). There were ambiguous or unsubstantiated implementation outcomes in a quarter (15/60, 25%) of the studies reviewed.

### Study Quality

Overall, study quality, as assessed using the MMAT, was low. A quarter of the reviewed studies (15/60, 25%) studies passed all 5 questions in the MMAT, 22% (13/60) of studies passed 4 questions, and 20% (12/60) of studies passed 3 questions. Overall, 12% (7/60) of studies passed 2 questions and 3% (2/60) of studies passed 1 question. Due to a lack of justification for the use of mixed methods and an absence of rigor in qualitative methods, 18% (11/60) of studies did not pass any of the 5 questions in the MMAT. There were clear research questions in 62% (37/60) of included studies, and 65% (39/60) appeared to have clear alignment between implementation aims and outcome measures. Finally, it was noted that studies often excluded individuals based on a range of comorbidities and sociocultural factors typically associated with ABI, and focused on the chronic stage of recovery (refer to *Domain 1: Condition* section). This presents a possible sampling bias, with some studies acknowledging limited generalizability. The higher proportion of implementation successes than failures may also indicate potential publication bias.

### Stakeholder Involvement

Formal input was obtained from people with ABI (33/60, 55%), clinicians (15/60, 25%), and caregivers (14/60, 23%) in some of the reviewed studies. However, this was overwhelmingly obtained during intervention evaluation (40/42, 95%) rather than development (16/42, 38%) stages. Rietdijk et al [44] informally incorporated qualitative feedback from the users of a previous iteration of the intervention into the intervention design, in addition to formally obtaining evaluation feedback. None of the reviewed studies (0/60, 0%) involved stakeholders in coproducing research, some did not involve stakeholders at all (13/60, 22%), and a minority (5/60, 8%) provided no information.

### Interventions

The most common intervention type was web-based education (10/60, 17%), including an intervention that combined education with cognitive rehabilitation [57]. Other interventions for which implementation data were available included cognitive exercises and games (8/60, 13%), Communication Partner Training (4/60, 7%), and Cognitive Behavioral Therapy (3/60, 5%). Interventions classified as *Other* (Multimedia Appendix 2; 35/60, 58%) included aphasia groups [42,43,58], emotional regulation training [54,59], and metacognitive rehabilitation [60,61]. Interventions were usually completely (26/60, 43%) or partly (21/60, 35%) individualized to the recipient’s specific needs and preferences. This individualization typically occurred via the clinician (35/47, 74%) or user-selected preference (30/47, 64%). Individualization also or instead occurred through automation, such as embedded logic or artificial intelligence (15/47, 32%). This was in contrast to generic interventions that were not modified according to individual needs and preferences (7/60, 12%). Some studies provided no information about the degree of individualization (6/60, 10%). Most studies included human interaction, primarily with the clinician (39/60, 65%); however, some studies provided opportunities for peer interaction among people with ABI (14/60, 23%) and among caregivers (4/60, 7%). Some interventions involved no interaction (12/60, 20%) or only interaction with artificial intelligence (9/60, 15%).

### The NASSS Framework

#### Domain 1: Condition

This review examined implementation evidence for interventions targeting adults with stroke (37/60, 62%), TBI (24/60, 40%), and aphasia of unspecified origin (4/60, 7%), or the formal (eg, clinicians and support workers) and informal (eg, family and partners) caregivers of this population. The psychosocial conditions under treatment included cognitive impairments, social communication difficulties, and language impairments among people with ABI and depression and caregiver burden among carers.

In each study, the nature of ABI and the psychosocial condition under treatment was almost always (56/60, 93%) *complicated* because it was “not fully characterized or understood” [17], yet not quite *complex* because participants were usually recruited in the chronic stages of injury to control for spontaneous recovery. In the remaining 7% (4/60) of studies, the condition shifted into the “unpredictable or high risk” definition of *complex* when investigators documented that participants experienced multiple neurological events [46,62,63] and responded unexpectedly to intervention during the chronic stage of injury [62,64].

Comorbidities and sociocultural factors were often (34/60, 57%) *simple* in studies because investigators set them as exclusion criteria. For example, investigators typically excluded participants with sensory or physical disabilities (including hemiparesis secondary to stroke), which would affect device use; people with intellectual disabilities; people with photosensitive epilepsy and other neurological conditions; people with mental illnesses or substance dependency; and people without carer support, device and computer proficiency, or internet access. These exclusions were acknowledged by some as a sampling bias. From among the 40% (24/60) of studies that considered or managed these comorbidities as *complicated* and *complex*, example considerations and learnings are provided in Textbox 1.

**Examples with citations from the reviewed studies of ways to accommodate common comorbidities.**

**Vision and hearing impairment [43]**

- Opportunity to perform training task with researcher. Multimodal training and intervention sessions, including both auditory and visual components. All training and intervention materials used at least 14-point font, white space, and clear images. Headset microphones were used to allow users to control volume, potentially reducing the impact of background noise [43].

**Cognition, memory, language, and attention [43,49,63,65,66]**

- Use of desktop shortcuts to enter videoconferencing software and aphasia-friendly training materials with significant use of white space [43].
- Explicit categorization; repetition of important units of information; use of plain language and text made suitable for Australian grade-5 reading age; and following health education guidelines for people with dysphasia, such as font size and number of words per screen [46].
- Uniformity in screen design regarding backgrounds, colors, and layout. Use of accessibility and usability guidelines. Use of simple interaction methods, such as mouse clicks on big buttons, to facilitate the possibility of using the same interface for touchscreen devices [49].

**Psychomotor, fine motor, and mobility [43,46,63]**

- Stroke survivors suggested that the program can be developed to take into account physical ability limitations or restrictions that were due to other comorbidities [46].
- Ensuring that software or processes did not require quick responses. Appropriate positioning of required equipment in initial training session to ensure access [43].
- Participants with hemiparesis were able to make required responses with their other hand [62].

#### Domain 2: Technology

Implementation evidence was included from web-based interventions on a range of devices including desktop computers (35/60, 58%), tablets (7/60, 12%), desktop computers and tablets (6/60, 10%), or desktop computers and mobile devices (tablet and smartphone; 6/60, 10%). Few interventions were both tablet and smartphone apps (2/60, 3%) or a solely smartphone app (2/60, 3%). Only 3% (2/60) of studies provided no information. Half of the reviewed interventions were delivered via telehealth videoconferencing (30/60, 50%). Interventions also used a combination of text (25/60, 42%), images (19/60, 32%), audio (13/60, 22%), and video (12/60, 20%); interactive games (15/60, 25%); virtual reality (2/60, 3%); productivity tools (eg, calendar and note-taking application; 4/60, 7%); and electronic communication systems, such as instant messaging (7/60, 12%), forums or message boards (5/60, 8%), and email (11/60, 18%). Reflecting the dominance of videoconferencing, interventions were frequently clinician-led (27/60, 45%). However, almost as many (23/60, 38%) interventions were completely automated or self-guided, reflecting the substantial proportion of cognitive exercises and games represented. Other interventions (10/60, 17%) were partly automated and partly telehealth.

The technology was often (37/60, 62%) a *simple* off-the-shelf or preinstalled solution, including hardware provision and software installation by the researchers. Similarly, the technology supply model was often off-the-shelf or a software requiring minimal customization (29/60, 48%). Approximately one-third of technologies were *complicated* in that they were not yet fully developed or interoperable (20/60, 33%), requiring significant customization or bespoke solutions (22/60, 37%). Some studies did not provide information on supply models (9/60, 15%) or the complexity of the technology (3/60, 5%).

Although some technologies required no support or only *simple* instructions for use (9/60, 15%), most technologies (42/60, 70%) required detailed initial training and ongoing troubleshooting support. Data were mostly self-entered (39/60, 65%), but also entered by the clinician (26/60, 43%) or automatically (18/60, 30%). A minority of studies (5/60, 8%) provided no information. Most often (29/60, 48%), these data were *complicated* in that they only “partially and indirectly measured changes in the condition” [17]. In some studies (9/60, 15%), the connection between data and the condition was *simple*, “directly and transparently” measuring change [17]. In other studies (8/60, 13%), it was *complex*, when the link between data generated and changes in the condition were “unpredictable or contested” [17]. Almost a quarter of the studies (14/60, 23%) provided no information.

#### Domain 3: Value Proposition

**Figure 4:**
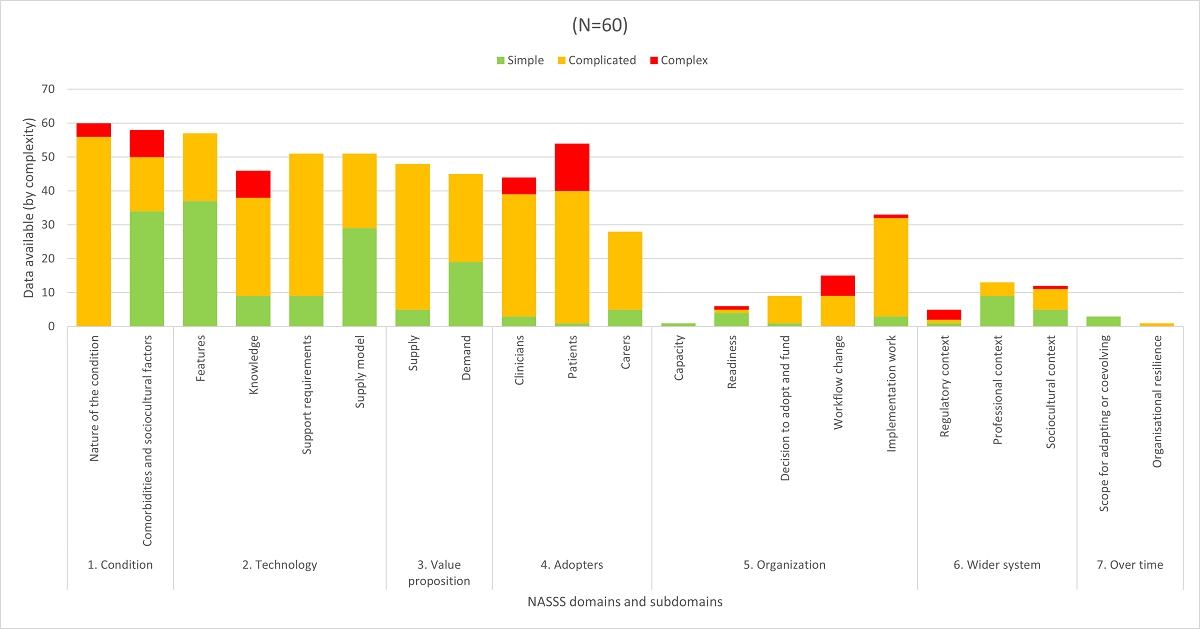
Quantification of complexity reported in each of the domains and subdomains of the Nonadoption, Abandonment, Scale-Up, Spread, and Sustainability (NASSS) framework in the included studies, showing (1) frequent controlling of comorbidities and sociocultural factors in domain 1; (2) contentious links between clinical changes in the condition and knowledge captured by the technology in domain 2; (3) particularly complex demands of patients and clinicians to use the interventions in domain 4; and (4) limited data in domains 5 to 7, documenting the challenge of workflow changes, a need for implementation work, and some complexity in relation to technology and regulations.

With the exception of only 3 studies [64,67,68], almost all (57/60, 95%) reviewed studies included some value proposition for their intervention, including a *supply-side* case (48/60, 80%). However, almost all *supply-side* cases (43/48, 90%) were *underdeveloped* [17]. Value propositions were more likely to be *simple* in relation to *demand-side* value to end users (Figure 4). Solana et al [49] uniquely demonstrated simplicity in both *demand-side* desirability to end users and a formally calculated *supply-side* economic benefit.

#### Domain 4: Adopters

Adopters included in this review included 5723 adults with ABI (5424, 95% intervention recipients and 299, 5% controls), 1920 formal and informal caregivers (1729, 90% recipients and 191, 10% controls), and 50 staff (4, 8% healthy recipients, such as volunteers, who trialed the intervention; 13, 26% administrative staff in 1 study [49]; and at least 33, 66% clinicians delivering interventions, as Anderson et al [69] did not specify how many clinicians were consulted). Reviewed interventions typically targeted only the person with ABI (44/60, 73%); however, some included both an informal carer and the person with ABI (7/60, 12%), informal carers only (6/60, 10%), or either formal or informal carers together with a person with ABI (2/60, 3%) [70,71]. None of the interventions targeted only formal caregivers (ie, clinicians or support workers), but Lee et al [72] (1/60, 2%) targeted both speech-language pathology students and people with ABI.

Of the 60 studies, there were 5 (8%) studies [44,45,47,67,68] reporting *complex* requirements of clinicians in terms of expanded or altered responsibilities and scope of practice. These included new implementation responsibilities to provide constant remote monitoring and troubleshooting [45,67,68] or overall resistance to the concept of telehealth with its perceived limitations in nonverbal communication and rapport building compared to face-to-face delivery [44,47]. *Complicated* involvement (36/60, 60%) required new training, skills, and personnel. Most obviously, synchronous telehealth interventions required clinicians to learn to deliver care via the internet rather than face-to-face, including videoconferencing, instant messaging, and web-based avatars. It also required them to at least be available for technical troubleshooting. Clinicians typically delivered therapy remotely from their own home, or a private office or clinic space. There were 3 studies (3/60, 5%) in which clinician involvement was not required [12,72,73] and 16 studies (16/60, 27%) provided no information.

Expectations of people with ABI were typically (53/60, 88%) high. Most were *complicated* (39/60, 65%), with minimum expectations to log on, enter data, and converse via the internet. Almost a quarter (14/60, 23%) of the reviewed studies described more *complex* requirements, such as reflective goal setting and adjustment in response to self-monitored progress. Only 1 intervention [55] *simply* targeted carers without involving individuals with ABI.

Although falling short of *complex* demands, carers were often (23/60, 38%) assumed or required to be available whenever needed, with requirements ranging from the provision of on-demand technical support to the person with ABI to participation in more intensive programs targeting the dyad or carer themselves. A minority of studies (5/60, 8%) reported that caregiver input was not required. However, more than half of the reviewed studies (32/60, 53%) provided no information about carer involvement.

#### Domain 5: Organization

All except 2 (58/60, 97%) studies [72,74] examined remote delivery to homes. A few had additional availability in community health (3/60, 5%), hospitals (4/60, 7%), or university or workplace settings (3/60, 5%). Implementation scope varied from single (20/60, 33%) and multiple sites (16/60, 27%) to state-wide (9/60, 15%), national (13/60, 22%), and—rarely—international (2/60, 3%) implementation.

The organizational role in implementation was mentioned in multiple studies (38/60, 63%). However, these data were sparse (Figure 4). The sole study [69] to specify an organization’s overall capacity to innovate described a progression in service delivery from face-to-face to a hybrid telephone, self-directed, and clinic-based service, to videoconference delivery using existing facilities.

A total of 6 studies (6/60, 10%) referred to organizational readiness for technological change. Readiness was made *simple* by the use of existing infrastructure [69,75], staff experience using technology with the clinical population [41], and organizational support for the shift [76]. However, readiness was *complicated* by workflow changes [63] and became *complex* in a public health system [77], where investigators anticipated challenges “relating to treatment space, technology availability or concerns about data protection,” but found the latter especially problematic due to internal regulations regarding approved information systems.

Of the reviewed studies, 15% (9/60) described an organization’s adoption and funding decision, which was rarely (1/9, 11%) *simple* [69], and most commonly (8/9, 89%) *complicated*, usually by partnerships with multiple organizations. Workflow changes were described in a quarter of the reviewed studies (15/60, 25%). None of the workflow changes were *simple*, and 40% (6/15) of studies reporting workflow change described them as *complex* [41,45,52,63,68,74] due to new demands on space, time, and skill (refer to *Domain 4: Adopters* section).

#### Domain 6: Wider System

Investigators mentioned the *wider system* of implementation in some studies (24/60, 40%), but data were again limited (Figure 4). The wider system was mostly mentioned in relation to the position of professional bodies (13/60, 22%), followed by comments on sociocultural factors (12/60, 20%) such as internet access and technological acceptance.

In addition, a minority of studies (5/60, 8%) commented on the regulatory context. While one study made passing note of potential barriers to billing telehealth [57], the regulatory context was primarily (4/5, 80%) described in relation to security. Data security was typically (3/4, 75%) *complex* [44,63,77], with health data considerations in relation to both bespoke and off-the-shelf platforms such as Facetime (Apple Inc.) and Skype (Microsoft Corporation). The only study where this subdomain was *simple* used a legally compliant platform [49].

The only explicit mention of the political context was in relation to the widespread use of telemedicine by the American Department of Veterans Affairs [57]. Studies from several countries acknowledged government funding sources, but no other political information was available beyond state and country names.

#### Domain 7: Embedding and Adaptation Over Time

A minority (4/60, 7%) of studies [41,47,69,78] included data on the seventh domain. Although 3 studies [41,47,78] described a strong scope for adapting and coevolving the intervention using end user input, no information on organizational resilience was available. Inversely, although Anderson et al [69] did not provide information on adapting the intervention, theirs was the sole study to document organizational resilience in managing unforeseen complications. These included increasing bandwidth allocation when it was insufficient in the organizational network and dispatching a research assistant to resolve ad hoc technical issues on-site.

### Complexity

A descriptive comparison of the records reporting successful implementation (37/45, 82%) and the those reporting failures (8/45, 18%) appears consistent with the following 3 hypotheses by Greenhalgh et al [20].

#### Hypothesis 1: If Most or All of the Domains Can Be Classified as Simple, an Intervention Is Likely to Be Easy to Implement and to Be Achieved on Time and Within Budget

If *most or all* were to be mathematically defined as 4 to 7 out of 7 domains, none of the reviewed studies met this definition. Therefore, it was not possible to confirm that an intervention with *most or all simple* domains was likely to succeed. Indeed, it was rare (4/60, 7%) to experience *simplicity* in >1 domain.

However, the mean number of *simple* domains and subdomains was higher for successes (mean 0.6 for domains and 3.3 for subdomains) than for *failures* (mean 0.1 for domains and 2.1 for subdomains), aligning with the possibility that more *simple* domains may increase the likelihood of implementation success.

Among failures, 88% (7/8) of the studies had no *simple* domains at all. There was only a single study (1/8, 13%) containing 1 *simple* domain. Among successes, 49% (18/37) had no *simple* domains, 43% (16/37) had 1 *simple* domain, 5% (2/37) had 2 *simple* domains, and 3% (1/37) had 3 *simple* domains. This also suggests that, even if it were true that an intervention with *most or all* simple domains is likely to succeed, the threshold for success may be much lower, and may necessarily be so, given that simplicity was so rare.

#### Hypothesis 2: If Many Domains Are Classified as Complicated, the Intervention Will Be Achievable but Difficult and Likely to Exceed Its Timescale and Budget

If *many domains* were to be defined as 3 to 7 domains, the hypothesis that implementation is still *achievable* with this many domains was supported; studies with as many as 6 (out of 7) *complicated* domains and 10 (out of 21) *complicated* subdomains still achieved implementation *success*. However, the hypothesis that implementation might be *difficult* could be reflected in that the degree of *complication* was similar for successes and failures at both the domain (mean 3.6 for successes and 3.4 for failures) and subdomain levels (mean 6.5 for successes and 6.6 for failures).

#### Hypothesis 3: If Multiple Domains Are Complex, the Chances of the Intervention Succeeding at All Are Limited

The mean number of complex domains was indeed higher among failures (mean 1.25 at both the domain and subdomain levels) than among successes (mean 0.8 at both the domain and subdomain levels). This appears to be consistent with the notion that more complex domains may hinder implementation.

## Discussion

### Principal Findings

In this review, we identified and appraised 60 published, peer-reviewed primary studies of implementation outcomes, strategies, or factors for web-based psychosocial interventions targeting either people with ABI, their formal or informal caregivers, or both. We narratively synthesized and quantified implementation data according to the NASSS framework to provide recommendations for future implementation. Recorded limitations of the evidence were the exclusion of individuals with a range of comorbidities and psychosocial challenges; poor methodological quality, particularly in the use of mixed methods; inconsistent use of implementation terminology; lack of theoretical underpinning; and limited data describing organizational, systemic, and long-term considerations. Our quantification of complexity across more than a decade of implementation evidence was consistent with all 3 of the following hypotheses: (1) *simplicity* facilitates implementation success; (2) successful implementation is more difficult, but still possible with more *complicated* domains; and (3) *complexity* makes implementation challenging and prone to failure [20]. These results align with recommendations to reduce complexity as much and in as many domains as possible to facilitate successful implementation [20].

### Complexity and Implementation

A complexity paradigm posits that digital health implementation has many domains of complexity and that they interact. This review is the first to quantify the specific domains in which complexity has occurred and identify potential targets to improve implementation. As seen in Figure 4, most complexity in the implementation of web-based interventions for people with ABI and their caregivers was reported in the intervention demands of people with ABI and their clinicians (domain 4); the ability of the technology to measure, convey, and enable responses to health data (domain 2; Figure 4); the changes introduced to clinical workflows (domain 5); and the need to manage health data regulations (domain 6). While confirming previous findings [10,15,26,27], a complexity paradigm provides new insight that these complexities may be counterbalanced by simplifying other domains and subdomains to enable implementation success. This included simplifying domain 1 (the condition) by excluding certain comorbidities and sociocultural factors and simplifying domain 2 (the technology) by selecting *off-the-shelf* products.

### The Importance of Intervention Value Propositions

In particular, a complexity paradigm highlights the relative simplicity observed in domain 3 (the value proposition) and the potential role of this simplicity in overall implementation success. Our results corroborate other digital health implementation reviews in finding that, despite their critical importance to sustainability [14], economic cases and business models for digital interventions were rarely articulated [22-24]. Financial viability remains a key challenge in digital health implementation [11], as the development and implementation of digital health interventions incur both up-front and ongoing costs. The low number of studies examining implementation beyond initially controlled studies may reflect overall sustainability challenges [11] in financially progressing past initial product development and testing [14]. Investigators seeking to improve the communication of this *supply-side* value proposition (domain 3) may benefit from both collaboration with stakeholders to identify and articulate an intervention’s economic value and interdisciplinary knowledge in health economics, business, and marketing. In the absence of such an economic case, a complexity perspective highlights that demonstrations of *demand-side* value, including measures of participant satisfaction, acceptability, and feasibility, become especially important to initially establish. The reviewed evidence primarily contributed to the value proposition in this subdomain, thus simplifying domain 3.

### Stakeholder Inclusion

The paradox is that *demand-side* value propositions were effectively undermined in the reviewed evidence by reductions in scope. Given the simplification of conditions (domain 1) is not possible in real-world settings, research participant exclusions based on specific comorbidities and sociocultural factors threaten the external validity of interventions [79] and reduce their potential reach due to an unrepresentative population. The exclusion of individuals with comorbidities can in fact disqualify the overwhelming majority of, and sometimes almost all, individuals with a target condition [79-81]. Such exclusions are therefore considered an increasingly untenable practice even in efficacy research, particularly given global population aging and increasing multimorbidity [80]. Continuation of this practice effectively creates a mismatch between the available evidence and clinical populations with ABI [81], the majority of which present with comorbidities [81] and psychosocial challenges [6-8,82]. Currently, this leaves clinicians and researchers ill-equipped to adequately understand how web-based psychosocial interventions may or may not be implemented with a real-world population.

Our finding of the simplification of domain 1 (the condition) corroborates reports in other systematic reviews of an unaddressed digital divide and noticeable lack of data pertaining to individuals with comorbidities in digital health implementation research more broadly [26,83]. In a recent review of digital mental health implementation [83], the most established category of digital psychosocial support [84], Barnett et al [83] discovered an absence of evidence pertaining to individuals with comorbidities and limited data on ethnic minorities. Therefore, researchers or practitioners seeking implementation data pertaining to the excluded conditions will need to seek implementation studies where such excluded conditions are the primary, rather than comorbid condition. For example, a recent systematic review of digital health implementation for individuals with psychosis or bipolar disorder [27] identified that the complexity of digital health interventions can be challenging for people with psychiatric symptoms. Given that our review identified similarly complex demands of interventions for individuals with a primary diagnosis of ABI (domain 4; Figure 4), it may be possible to see how *comorbid* psychosis and ABI diagnoses can create further complex interactions between domains 1 (the condition) and 4 (the adopters) that were not identified in the reviewed evidence.

The underrepresentation of clinical complexities also raises ethical questions of equity [26,83] in the development of interventions for people with ABI. If no interventions are designed or tested with these excluded populations, there is a risk of perpetuating and exacerbating the disadvantages experienced by individuals already at greater risk of digital exclusion [26,83]. In the words of one of our research collaborators, Mrs Erin Elizabeth Hill, who has living experience of ABI:

If your aim is to help people with ABI, then you can’t exclude a whole group of us, when there are more of us that have these conditions than don’t. You can’t say, “Oh, we considered ‘some’ of you.” You need to be as inclusive as possible.

Similar reviews in other populations have concluded that there is an urgent need for data pertaining to marginalized groups in digital health implementation research [26,83]. Sampling and publication biases across a body of reviewed evidence risk reducing the visibility of individuals who can be “forgotten when taking the findings of this review into consideration” [26]. Therefore, it may be helpful for researchers and clinicians to be mindful of this gap at the study design stage [79,85], not only to facilitate real-world implementation, but also to ensure that future digital health intervention design, implementation, and research does not create and perpetuate inequities [26,83].

Investigators who made proactive efforts to maximize inclusion in the reviewed studies provided valuable data about how real-world implementation may or may not be achieved. In a study that originally excluded people with ABI who had a history of falls [61], researchers recognized during recruitment that such a history was common in the population that may benefit from the intervention. They subsequently adjusted their screening criteria, based on expert panel advice, to focus on self-awareness rather than fall history, enabling previously excluded people with ABI to participate. In another study that accommodated medical complexity [63], investigators documented how readmission for subsequent strokes influenced a person with ABI’s adherence to a tablet-based intervention. Although the participant eventually discontinued use, implementation data concerning dropouts and reasons for discontinuation contribute an important real-world understanding of implementation. Moreover, they enabled people with ABI who wished to participate in research and receive interventions to do so to the extent that they were able. This illustrates how, in addition to empirical questions of external validity and ethical questions of equity, population exclusions may imply a divergence between the priorities and realities of researchers and stakeholders [21], thus requiring a priori effort if it is to be overcome.

### Stakeholder Collaboration

Facilitating implementation despite the real-world complexity of ABI (domain 1) presents researchers with the challenge of simplifying as many of the remaining domains (domains 2-7) as possible. Direct collaboration with stakeholders may be key to this endeavor, over and above inclusion in participant samples. In this review, population disparities were magnified by the consultation of the already unrepresentative study populations during intervention evaluation rather than development, with no studies coproduced with stakeholders. However, it is frequently recommended that stakeholders are engaged from the outset of research, rather than only in intervention evaluation [27,85-87]. To support similar efforts, we have previously published [21,34] methodological guidance on how to leverage the NASSS framework to facilitate implementation input from people with ABI, their clinicians and close others.

The need for stakeholder collaboration is further supported by our finding that the most complexity was reported in the interventional and technological requirements of people with ABI and clinicians in domain 4 (Figure 4). Simplifying an intervention’s demands of people with ABI presents the largest current target to reduce complexity, with stakeholder collaboration providing opportunity to identify ways to accommodate comorbidities (domain 1; Textbox 1), co-design (domain 4), and continue to streamline (domain 7) interventions. Additionally, digital health interventions introduce complex new demands on clinicians (domain 4; Figure 4), including many tasks specific to digital health, such as remote monitoring, intervention adjustment, and providing and receiving ongoing technical support. It also introduces new space, equipment, and privacy requirements for telehealth sessions, with subsequent challenges at the organizational level (domain 5). These are critical considerations given the importance of workflow in the success or failure of digital health implementation [15]. In particular, the role of carers was underreported in this review, and only a single study [49] included the input of administrative staff who may be required to support or implement these functions, suggesting that these stakeholder groups are less visible and consulted. Additional implementation work and the staff who will likely undertake such work (domain 5; Figure 4) can also be easily overlooked due to both limited data at the organizational level (domain 5) and potential for investigators to absorb implementation work in the context of a research study. Therefore, our findings highlight the importance of recognizing health care staff adopters as stakeholders. Again, these collaborations will require significant resources and funding [88], indicating a need for researchers to upskill in the communication of value propositions to funders (domain 3) and for funders to create and invest in in the resource-intensive process of stakeholder collaboration.

### Theoretical Frameworks

Finally, it is recommended that investigators use implementation frameworks, and specifically digital health implementation frameworks such as user-centered design or the NASSS framework, to underpin implementation research. User-centered design may be especially pertinent given a need to simplify domain 4 (Figure 4). The NASSS framework may also facilitate consideration across multiple domains. Our results aligned with the NASSS framework’s wide-ranging inclusion of considerations in digital health implementation, with the reviewed evidence revealing challenges in all 7 domains. Our findings were consistent with other reviews applying the NASSS framework [22-24] and those using generic implementation frameworks [26-29] in revealing a general lack of implementation evidence at the organizational level and beyond (domains 5-7; Figure 4). This may reflect the concurrent finding of limited theoretical underpinning in the reviewed studies. The limited data on organizational, systemic, and long-term aspects of implementation in this and other reviews reveal a significant gap in research evidence to date, which should be addressed to support the ecological validity and implementation of future interventions.

### Study Strengths and Limitations

Reviews to date have relied on narrative syntheses when examining digital health implementation [28] and complexity [22-24]. This review was the first to both deductively analyze [25] the evidence in relation to each of the 7 domains of a complexity-based framework and quantify complexity across more than a decade of digital health implementation data against published hypotheses [20]. This investigation has enabled new understanding of the complex interrelationships between domains.

This registered review followed a published protocol [30], with deviations and rationales that are reported transparently. All extracted data, search strategies, and extraction forms are transparently appended, and results are reported according to PRISMA 2020 guidelines. To further increase replicability, data were consistently extracted and appraised using published definitions [36], taxonomies [38], and tools [37]. Although other reviews have not included information on stakeholder involvement [26] and used generic implementation frameworks [26-28], our search and synthesis were both informed by stakeholder input [21] and theoretically underpinned by an implementation framework specific to digital health [17]. Unlike reviews that focused more exclusively on scale-up [24] or implementation strategies [11], this review included and classified a wide range of data on implementation factors, outcomes, and strategies. The inclusiveness of our search thus allowed previously unreviewed implementation data to be included for the first time.

The high yield and detailed extraction from >21 theoretical subdomains presented substantial feasibility challenges, requiring pragmatic protocol deviations. Extraction was challenging due to the heterogeneity and inconsistent reporting of interventions and implementation outcomes. For instance, Pierce and Steiner [89] collectively reported satisfaction, acceptability, usability, and feasibility measures as *usability*, and Anderson et al [69] measured *satisfaction* using feasibility and acceptability measures. Therefore, standardized definitions [36] were required in the extraction form (Multimedia Appendix 2). Implementation evidence also has limited alignment with the clinical paradigm of PRISMA guidelines. Given our need for an innovative, theory-based meta-synthesis and quantification of complexity, an implementation-specific review checklist with a complexity paradigm may need to be developed as scientific understanding of both implementation and complexity grows.

As our review examined prepandemic evidence published up to June 2020, it will be possible in future to compare COVID-related and postpandemic evidence with our findings. Given that the global COVID-19 pandemic has brought domain 6 to the fore, a future update of our review that allows sufficient time for implementation effort and publication from these periods may offer a unique opportunity to meta-synthesize and directly compare data from 2 distinct epochs, allowing scrutiny of the impact of national and international shifts in this domain.

A key limitation of the reviewed evidence was that the overall quality of reviewed studies was low, with challenges for external validity due to study populations that were unrepresentative of the real-world population with ABI. The resulting potential for sampling bias may reflect pressures upon researchers to establish a *demand-side* value proposition in domain 3. The high proportion of successes may also indicate possible publication bias, which may be exacerbated by the same pressure. Another limitation was that the English-speaking research team was restricted to English publications. Finally, although the results of this descriptive analysis appear consistent with all 3 hypotheses by Greenhalgh et al [20], definitive relationships could not yet be established because:

1. The sample size of this study was not adequately powered to detect an association between complexity and implementation success or failure. In particular, the limited number of failures available for review may reflect (1) the controlled nature of clinical trials, which inherently aim to minimize complexity and (2) potential publication bias toward reporting implementation success. The intensive process of deductively coding 21 subdomains of complexity in a highly heterogeneous evidence base also reduced the feasibility of obtaining a sufficiently large sample size.
2. Although some studies reported information concerning all 7 domains of complexity, none of the studies reported data from all subdomains. Information about complexity was thus incomplete for all records.
3. In the absence of a formal definition [11], implementation success or failure of each study was defined as whether the authors succeeded in achieving their specific implementation aims, which varied in scope and type (eg, usability, cost-effectiveness, and satisfaction are distinct constructs). Studies generally also did not report planned or actual budgets and timescales as described in the hypotheses by Greenhalgh et al [20].

Given these limitations, the eventual alignment between a flexible definition of success and all 3 hypotheses [20] was unexpected. It may suggest some correlation between the various implementation measures. For example, a highly appropriate intervention may also be more acceptable to recipients, receive high satisfaction ratings, and experience increased adherence and fidelity from motivated actors. This, in turn, may improve its feasibility and cost-effectiveness. Alternatively, or perhaps concurrently, the concept of complexity may be informative for a range of implementation constructs, with further research needed to understand these relationships. For example, a multiarm RCT comparing different levels of complexity might assist our understanding of complexity within implementation science [90]. Parallel reviews of digital health implementation for other health conditions and intervention types (eg, health informatics) may also be informative; we have transparently supplied our search terms (Multimedia Appendix 1) and extraction template (Multimedia Appendix 2) to support this endeavor.

### Conclusions

This study is the first attempt to deductively analyze and quantify complexity from more than a decade of primary digital health implementation evidence. Results were consistent with recommendations to facilitate implementation success by reducing complexity in as many domains as possible. To date, simplifications appear to have been made in the first domain of the NASSS framework (the condition) to advance the value proposition of interventions (domain 3). However, this may hinder the development of equitable, real-world solutions for which implementation data and end user support are currently needed. It is recommended that intervention developers collaborate with stakeholders, including people with ABI, their close others, and clinicians, from the earliest design stages, rather than only at end-evaluation, to target real-world complexities in domains 2 to 7. Recommended future research includes parallel reviews for other populations and intervention types, multiarm RCTs to test the role of complexity in digital health implementation, and reviews of evidence obtained during or after the COVID-19 pandemic to understand the impact of the wider context of digital health implementation (domain 6).

## Appendix

Multimedia Appendix 1 Search strategy.

Multimedia Appendix 2 REDCap (Research Electronic Data Capture) extraction form.

Multimedia Appendix 3 Extracted data and analysis of complexity.

## Abbreviations

ABI: acquired brain injury
MMAT: Mixed Methods Appraisal Tool
NASSS: Nonadoption, Abandonment, Scale-Up, Spread, and Sustainability
PRISMA: Preferred Reporting Items for Systematic Reviews and Meta-Analyses
PROSPERO: International Prospective Register of Systematic Reviews
RCT: randomized controlled trial
REDCap: Research Electronic Data Capture
TBI: traumatic brain injury

## Data Availability

The search strategy is available as Multimedia Appendix 1, data extraction form is included as Multimedia Appendix 2, and data extracted from included studies and used for analyses are provided as Multimedia Appendix 3.
