# Supplementary material for: Implementation of Web-Based Psychosocial Interventions for Adults With Acquired Brain Injury and Their Caregivers: Systematic Review
Source: J Med Internet Res. 2022 Jul 26;24(7):e38100. doi: 10.2196/38100 (PMC9328122; doi:10.2196/38100)
Supplement: Multimedia Appendix 1 [file jmir_v24i7e38100_app1.pdf]

Search Strategy in each database

1. Scopus

The following search was run:

((TITLE-ABS-KEY (implement* OR effectiveness-implementation OR disseminat* OR diffus* OR utili* OR sustainab* OR facilitat* OR barrier* OR scalab* OR "Process evaluation" OR "process measure*" OR feasib* OR adopt* OR adapt* OR uptake* OR usab* OR "lessons learned" OR implications OR experiences OR interoperab* OR fail* OR succes* OR acceptab* OR appropriate* OR fidelity OR ahere* OR complian* OR penetration OR cost* OR satisfaction OR hybrid OR pragmatic OR co-design OR codesign OR participatory* OR ((knowledge OR research OR technology) AND (translat* OR transform* OR exchange OR transfer OR integration OR utili?ation))))) AND ((TITLE-ABS-KEY (telehealth OR tele-health OR telepractice OR tele-practice OR "digital therapy" OR mhealth OR m-health OR "health telematics" OR telemedicine OR tele-medicine OR computeri?ed OR internet-delivered OR internet-based OR online OR web-based OR "digital health" OR e-health OR ehealth OR "wireless health" OR e-therapy OR etherapy OR "healthcare technology" OR e-rehabilitation OR erehabilitation OR "User-Computer Interface" OR "digital therapeutic*" OR e-mental OR emental OR "digital mental health"))) AND (((TITLE-ABS-KEY ("brain injury" OR neurotrauma OR tbi OR "Traumatic brain injury" OR "head injury" OR "stroke" OR "cerebrovascular accident" OR cva OR aphasia OR dysphasia OR "primary progressive aphasia" OR dementia OR parkinson* OR alzheimer* OR migraine OR meningitis OR "brain cancer" or "brain tumour" or "brain tumor" or "brain neoplasm*" or encephalitis or tetanus or epilepsy)))) AND (((TITLE-ABS-KEY ("brain injury" OR neurotrauma OR tbi OR "Traumatic brain injury" OR "head injury" OR "stroke" OR "cerebrovascular accident" OR cva OR aphasia OR dysphasia OR "primary progressive aphasia" OR dementia OR parkinson* OR alzheimer* OR migraine OR meningitis OR "brain cancer" or "brain tumour" or "brain tumor" or "brain neoplasm*" or encephalitis or tetanus or epilepsy)))) AND (LIMIT-TO (PUBYEAR,2020) OR LIMIT-TO (PUBYEAR,2019) OR LIMIT-TO (PUBYEAR,2018) OR LIMIT-TO (PUBYEAR,2017) OR LIMIT-TO (PUBYEAR,2016) OR LIMIT-TO (PUBYEAR,2015) OR LIMIT-TO (PUBYEAR,2014) OR LIMIT-TO (PUBYEAR,2013) OR LIMIT-TO (PUBYEAR,2012) OR LIMIT-TO (PUBYEAR,2011) OR LIMIT-TO (PUBYEAR,2010) OR LIMIT-TO (PUBYEAR,2009) OR LIMIT-TO (PUBYEAR,2008)) AND (LIMIT-TO (LANGUAGE,"English")) AND (LIMIT-TO (DOCTYPE,"ar") OR LIMIT-TO (DOCTYPE,"re")) AND (LIMIT-TO (SRCTYPE,"j"))

2. Medline via Ovid

Set	Search Statement
1.	implement*.mp.
2.	effectiveness-implementation.mp.
3.	disseminat*.mp.
4.	(diffusion adj2 innovation).mp. [mp=title, abstract, original title, name of substance word, subject heading word, floating sub-heading word, keyword heading word, organism supplementary concept word, protocol supplementary concept word, rare disease supplementary concept word, unique identifier, synonyms]
5.	utili*.mp.
6.	sustainable development/ or "health care (non mesh)"/ or "health care facilities, manpower, and services"/ or "health care economics and organizations"/ or health services administration/ or "health care quality, access, and evaluation"/
7.	uptake*.mp.
8.	facilitat*.mp.
9.	barrier*.mp.
10.	scalab*.mp. [mp=title, abstract, original title, name of substance word, subject heading word, floating sub-heading word, keyword heading word, organism supplementary concept word, protocol supplementary concept word, rare disease supplementary concept word, unique identifier, synonyms]
11.	"process measure*".mp. [mp=title, abstract, original title, name of substance word, subject heading word, floating sub-heading word, keyword heading word, organism supplementary concept word, protocol supplementary concept word, rare disease supplementary concept word, unique identifier, synonyms]
12.	"process evaluation".mp. [mp=title, abstract, original title, name of substance word, subject heading word, floating sub-heading word, keyword heading word, organism supplementary concept word, protocol supplementary concept word, rare disease supplementary concept word, unique identifier, synonyms]

13.	"outcome and process assessment, health care"/ or process assessment, health care/
14.	exp Feasibility Studies/ or feasib*.mp.
15.	adopt*.mp.
16.	adapt*.mp.
17.	usab*.mp.
18.	"lessons learned".mp.
19.	implications.mp.
20.	experiences.mp.
21.	interoperab*.mp. [mp=title, abstract, original title, name of substance word, subject heading word, floating sub-heading word, keyword heading word, organism supplementary concept word, protocol supplementary concept word, rare disease supplementary concept word, unique identifier, synonyms]
22.	fail*.mp.
23.	success*.mp.
24.	acceptab*.mp. [mp=title, abstract, original title, name of substance word, subject heading word, floating sub-heading word, keyword heading word, organism supplementary concept word, protocol supplementary concept word, rare disease supplementary concept word, unique identifier, synonyms]
25.	appropriate*.mp.
26.	fidelity*.mp.
27.	adhere*.mp. [mp=title, abstract, original title, name of substance word, subject heading word, floating sub-heading word, keyword heading word, organism supplementary concept word, protocol supplementary concept word, rare disease supplementary concept word, unique identifier, synonyms]

28.	complian*.mp. [mp=title, abstract, original title, name of substance word, subject heading word, floating sub-heading word, keyword heading word, organism supplementary concept word, protocol supplementary concept word, rare disease supplementary concept word, unique identifier, synonyms]
29.	penetration.mp.
30.	cost*.mp. or exp "Costs and Cost Analysis"/
31.	satisfaction.mp.
32.	hybrid.mp.
33.	exp Pragmatic Clinical Trials as Topic/ or exp Pragmatic Clinical Trial/ or pragmatic.mp.
34.	(co-design or codesign).mp. [mp=title, abstract, original title, name of substance word, subject heading word, floating sub-heading word, keyword heading word, organism supplementary concept word, protocol supplementary concept word, rare disease supplementary concept word, unique identifier, synonyms]
35.	exp "Treatment Adherence and Compliance"/
36.	exp Community-Based Participatory Research/ or participatory*.mp.
37.	((knowledge or research or technology) and (translat* or transform* or exchange or transfer or integration or utili#ation)).mp. [mp=title, abstract, original title, name of substance word, subject heading word, floating sub-heading word, keyword heading word, organism supplementary concept word, protocol supplementary concept word, rare disease supplementary concept word, unique identifier, synonyms]
38.	1 or 2 or 3 or 4 or 5 or 6 or 7 or 8 or 9 or 10 or 12 or 13 or 14 or 15 or 16 or 17 or 18 or 19 or 20 or 21 or 22 or 23 or 24 or 25 or 26 or 29 or 30 or 31 or 32 or 33 or 34 or 35 or 36 or 37
39.	limit 38 to yr="2008 -Current"
40.	limit 39 to english language
41.	telemedicine/ or telerehabilitation/
42.	telepractice.mp. [mp=title, abstract, original title, name of substance word, subject heading word, floating sub-heading word, keyword heading word, organism supplementary concept word, protocol supplementary concept word, rare disease supplementary concept word, unique identifier, synonyms]

43.	tele-practice.mp. [mp=title, abstract, original title, name of substance word, subject heading word, floating sub-heading word, keyword heading word, organism supplementary concept word, protocol supplementary concept word, rare disease supplementary concept word, unique identifier, synonyms]
44.	(telemedicine or tele-medicine).mp. [mp=title, abstract, original title, name of substance word, subject heading word, floating sub-heading word, keyword heading word, organism supplementary concept word, protocol supplementary concept word, rare disease supplementary concept word, unique identifier, synonyms]
45.	(mhealth or m-health).mp. [mp=title, abstract, original title, name of substance word, subject heading word, floating sub-heading word, keyword heading word, organism supplementary concept word, protocol supplementary concept word, rare disease supplementary concept word, unique identifier, synonyms]
46.	(telehealth or tele-health).mp. [mp=title, abstract, original title, name of substance word, subject heading word, floating sub-heading word, keyword heading word, organism supplementary concept word, protocol supplementary concept word, rare disease supplementary concept word, unique identifier, synonyms]
47.	computeri#ed.mp. [mp=title, abstract, original title, name of substance word, subject heading word, floating sub-heading word, keyword heading word, organism supplementary concept word, protocol supplementary concept word, rare disease supplementary concept word, unique identifier, synonyms]
48.	Internet-Based Intervention/
49.	internet-delivered.mp.
50.	internet-based.mp.
51.	online.mp.
52.	web-based.mp.
53.	"digital health".mp.
54.	(ehealth or e-health).mp. [mp=title, abstract, original title, name of substance word, subject heading word, floating sub-heading word, keyword heading word, organism supplementary concept word, protocol supplementary concept word, rare disease supplementary concept word, unique identifier, synonyms]
55.	"wireless health".mp.

56.	(etherapy or e-therapy).mp. [mp=title, abstract, original title, name of substance word, subject heading word, floating sub-heading word, keyword heading word, organism supplementary concept word, protocol supplementary concept word, rare disease supplementary concept word, unique identifier, synonyms]
57.	"healthcare technology".mp.
58.	(erehabilitation or e-rehabilitation).mp. [mp=title, abstract, original title, name of substance word, subject heading word, floating sub-heading word, keyword heading word, organism supplementary concept word, protocol supplementary concept word, rare disease supplementary concept word, unique identifier, synonyms]
59.	"digital therapeutic*".mp.
60.	"digital therapy".mp.
61.	(emental or e-mental).mp. [mp=title, abstract, original title, name of substance word, subject heading word, floating sub-heading word, keyword heading word, organism supplementary concept word, protocol supplementary concept word, rare disease supplementary concept word, unique identifier, synonyms]
62.	user-computer interface/
63.	41 or 44 or 45 or 46 or 47 or 48 or 49 or 50 or 51 or 52 or 53 or 54 or 55 or 56 or 57 or 58 or 59 or 60 or 61 or 62
64.	limit 63 to yr="2008 -Current"
65.	limit 64 to english language
66.	cognitive therapy.mp. or exp Cognitive Behavioral Therapy/
67.	exp Behavior Therapy/ or "behavio?r therapy".mp.
68.	(psychoeducation or psycho-education).mp. [mp=title, abstract, original title, name of substance word, subject heading word, floating sub-heading word, keyword heading word, organism supplementary concept word, protocol supplementary concept word, rare disease supplementary concept word, unique identifier, synonyms]
69.	((psychosocial or psycholog* or group or dyad* or family or caregiver or "communication partner" or communication) and (therapy or intervention or training or treatment or program or rehabilitation or coaching or education or support)).mp. [mp=title, abstract, original title, name of substance word, subject heading word, floating sub-heading word, keyword heading word, organism supplementary concept word, protocol supplementary concept word, rare disease supplementary concept word, unique identifier, synonyms]

70.	66 or 67 or 68 or 69
71.	limit 70 to yr="2008 -Current"
72.	limit 71 to english language
73.	38 and 63 and 70
74.	limit 73 to yr="2008 -Current"
75.	limit 74 to english language
76.	"traumatic brain injury".mp. or exp Brain Injuries, Traumatic/
77.	neurotrauma.mp.
78.	tbi.mp.
79.	stroke.mp. or exp Stroke/
80.	Cerebrovascular Trauma/rh, th [Rehabilitation, Therapy]
81.	exp Brain Injuries/ or "brain injur*".mp.
82.	"brain tumo?r".mp. or Brain Neoplasms/
83.	"brain cancer".mp.
84.	aphasia.mp. or exp Aphasia/ or exp Aphasia, Primary Progressive/
85.	dysphasia.mp.
86.	exp Alzheimer Disease/ or alzheimer*.mp.
87.	dementia.mp. or exp Dementia/
88.	parkinson*.mp. or exp Parkinson Disease/
89.	"motor neuron? disease".mp. or exp Motor Neuron Disease/

90.	"multiple sclerosis".mp. or exp Multiple Sclerosis/
91.	migraine.mp. or exp Migraine Disorders/
92.	meningitis.mp. or exp Meningitis/
93.	exp Encephalitis/ or encephalitis.mp.
94.	exp Tetanus/ or tetanus.mp.
95.	exp Epilepsy/ or epilepsy.mp.
96.	76 or 77 or 78 or 79 or 80 or 81 or 82 or 83 or 84 or 85 or 86 or 87 or 88 or 89 or 90 or 91 or 92 or 93 or 94 or 95
97.	38 and 63 and 70 and 96
98.	limit 97 to (english language and yr="2008 -Current")

3. Embase via Ovid

Set	Search Statement
1.	implement*.mp.
2.	effectiveness-implementation.mp.
3.	disseminat*.mp.
4.	(diffusion adj2 innovation).mp.
5.	utili*.mp.
6.	sustainab*.mp.
7.	uptake*.mp.
8.	facilitat*.mp.
9.	barrier*.mp.
10.	scalab*.mp.
11.	"process evaluation".mp.
12.	"process measure*".mp. [mp=title, abstract, heading word, drug trade name, original title, device manufacturer, drug manufacturer, device trade name, keyword, floating subheading word, candidate term word]
13.	exp feasibility study/ or feasib*.mp.
14.	adopt*.mp.
15.	adapt*.mp.
16.	exp computer interface/ or usab*.mp.
17.	"lessons learned".mp. [mp=title, abstract, heading word, drug trade name, original title, device manufacturer, drug manufacturer, device trade name, keyword, floating subheading word, candidate term word]
18.	implications.mp. [mp=title, abstract, heading word, drug trade name, original title, device manufacturer, drug manufacturer, device trade name, keyword, floating subheading word, candidate term word]
19.	experiences.mp. [mp=title, abstract, heading word, drug trade name, original title, device manufacturer, drug manufacturer, device trade name, keyword, floating subheading word, candidate term word]
20.	interoperab*.mp. [mp=title, abstract, heading word, drug trade name, original title, device manufacturer, drug manufacturer, device trade name, keyword, floating subheading word, candidate term word]
21.	fail*.mp. [mp=title, abstract, heading word, drug trade name, original title, device manufacturer, drug manufacturer, device trade name, keyword, floating subheading word, candidate term word]
22.	success*.mp. [mp=title, abstract, heading word, drug trade name, original title, device manufacturer, drug manufacturer, device trade name, keyword, floating subheading word, candidate term word]

23.	acceptab*.mp. [mp=title, abstract, heading word, drug trade name, original title, device manufacturer, drug manufacturer, device trade name, keyword, floating subheading word, candidate term word]
24.	appropriate*.mp. [mp=title, abstract, heading word, drug trade name, original title, device manufacturer, drug manufacturer, device trade name, keyword, floating subheading word, candidate term word]
25.	fidelity.mp. [mp=title, abstract, heading word, drug trade name, original title, device manufacturer, drug manufacturer, device trade name, keyword, floating subheading word, candidate term word]
26.	adhere*.mp. [mp=title, abstract, heading word, drug trade name, original title, device manufacturer, drug manufacturer, device trade name, keyword, floating subheading word, candidate term word]
27.	complian*.mp. [mp=title, abstract, heading word, drug trade name, original title, device manufacturer, drug manufacturer, device trade name, keyword, floating subheading word, candidate term word]
28.	penetration.mp. [mp=title, abstract, heading word, drug trade name, original title, device manufacturer, drug manufacturer, device trade name, keyword, floating subheading word, candidate term word]
29.	cost*.mp. [mp=title, abstract, heading word, drug trade name, original title, device manufacturer, drug manufacturer, device trade name, keyword, floating subheading word, candidate term word]
30.	satisfaction.mp. [mp=title, abstract, heading word, drug trade name, original title, device manufacturer, drug manufacturer, device trade name, keyword, floating subheading word, candidate term word]
31.	hybrid.mp. [mp=title, abstract, heading word, drug trade name, original title, device manufacturer, drug manufacturer, device trade name, keyword, floating subheading word, candidate term word]
32.	pragmatic.mp. [mp=title, abstract, heading word, drug trade name, original title, device manufacturer, drug manufacturer, device trade name, keyword, floating subheading word, candidate term word]
33.	co-design.mp. [mp=title, abstract, heading word, drug trade name, original title, device manufacturer, drug manufacturer, device trade name, keyword, floating subheading word, candidate term word]
34.	codesign.mp. [mp=title, abstract, heading word, drug trade name, original title, device manufacturer, drug manufacturer, device trade name, keyword, floating subheading word, candidate term word]
35.	participatory*.mp. [mp=title, abstract, heading word, drug trade name, original title, device manufacturer, drug manufacturer, device trade name, keyword, floating subheading word, candidate term word]
36.	((knowledge or research or technology) and (translat* or transform* or exchange or transfer or integration or utili#ation)).af.
37.	1 or 2 or 3 or 4 or 5 or 6 or 7 or 8 or 9 or 10 or 11 or 13 or 14 or 15 or 16 or 17 or 18 or 19 or 20 or 21 or 22 or 23 or 24 or 25 or 28 or 29 or 30 or 31 or 32 or 33 or 34 or 35 or 36
38.	telemedicine/ or telehealth/ or teleconsultation/ or telediagnosis/ or telerehabilitation/ or teletherapy/
39.	mhealth.mp.
40.	m-health.mp.
41.	telehealth.mp.
42.	tele-health.mp.

43.	telemedicine.mp. [mp=title, abstract, heading word, drug trade name, original title, device manufacturer, drug manufacturer, device trade name, keyword, floating subheading word, candidate term word]
44.	tele-medicine.mp. [mp=title, abstract, heading word, drug trade name, original title, device manufacturer, drug manufacturer, device trade name, keyword, floating subheading word, candidate term word]
45.	computeri#ed.mp. [mp=title, abstract, heading word, drug trade name, original title, device manufacturer, drug manufacturer, device trade name, keyword, floating subheading word, candidate term word]
46.	telepractice.mp. [mp=title, abstract, heading word, drug trade name, original title, device manufacturer, drug manufacturer, device trade name, keyword, floating subheading word, candidate term word]
47.	tele-practice.mp. [mp=title, abstract, heading word, drug trade name, original title, device manufacturer, drug manufacturer, device trade name, keyword, floating subheading word, candidate term word]
48.	internet-delivered.mp.
49.	internet-based.mp.
50.	online.mp.
51.	web-based.mp.
52.	"digital health".mp.
53.	e-health.mp.
54.	ehealth.mp.
55.	"wireless health".mp.
56.	e-therapy.mp.
57.	etherapy.mp.
58.	"healthcare technology".mp.
59.	e-rehabilitation.mp.
60.	erehabilitation.mp. [mp=title, abstract, heading word, drug trade name, original title, device manufacturer, drug manufacturer, device trade name, keyword, floating subheading word, candidate term word]
61.	"digital therapy".mp. [mp=title, abstract, heading word, drug trade name, original title, device manufacturer, drug manufacturer, device trade name, keyword, floating subheading word, candidate term word]
62.	"digital therapeutic*".mp.
63.	e-mental.mp.
64.	emental.mp.
65.	"digital mental health".mp.
66.	38 or 39 or 40 or 41 or 42 or 43 or 44 or 45 or 48 or 49 or 50 or 51 or 52 or 53 or 54 or 55 or 56 or 57 or 58 or 59 or 60 or 62 or 63 or 64 or 65
67.	exp cognitive therapy/ or exp cognitive behavioral therapy/ or exp behavior therapy/

68.	"behavio?r therapy".mp.
69.	psychology/ or psychological aspect/ or psycholog*.mp.
70.	psychoeducation*.mp. or psychoeducation/
71.	psycho-education*.mp.
72.	education/ or education.mp. or patient education/ or adult education/ or health education/ or education program/
73.	communication.mp. or exp interpersonal communication/
74.	((psychosocial or psycholog* or dyad* or group or family or caregiver or "communication partner" or communication) and (therapy or intervention or training or treatment or program or rehabilitation or coaching or education or support)).af.
75.	67 or 68 or 69 or 70 or 71 or 72 or 73 or 74
76.	traumatic brain injury.mp. or traumatic brain injury/
77.	stroke.mp. or exp cerebrovascular accident/
78.	"brain injury".mp. or exp brain injury/
79.	exp head injury/ or head injur*.mp.
80.	cva.mp.
81.	tbi.mp.
82.	neurotrauma.mp.
83.	exp Aphasia/
84.	exp Dementia/
85.	(aphasia or dysphasia).mp. [mp=title, abstract, heading word, drug trade name, original title, device manufacturer, drug manufacturer, device trade name, keyword, floating subheading word, candidate term word]
86.	dementia.mp. [mp=title, abstract, heading word, drug trade name, original title, device manufacturer, drug manufacturer, device trade name, keyword, floating subheading word, candidate term word]
87.	exp Aphasia, Primary Progressive/
88.	"Primary Progressive Aphasia".mp. [mp=title, abstract, heading word, drug trade name, original title, device manufacturer, drug manufacturer, device trade name, keyword, floating subheading word, candidate term word]
89.	alzheimer*.mp. [mp=title, abstract, heading word, drug trade name, original title, device manufacturer, drug manufacturer, device trade name, keyword, floating subheading word, candidate term word]
90.	exp Alzheimer Disease/
91.	exp Parkinson Disease/
92.	parkinson*.mp. [mp=title, abstract, heading word, drug trade name, original title, device manufacturer, drug manufacturer, device trade name, keyword, floating subheading word, candidate term word]
93.	motor neuron? disease.mp. [mp=title, abstract, heading word, drug trade name, original title, device manufacturer, drug manufacturer, device trade name, keyword, floating subheading word, candidate term word]

94.	exp Motor Neuron Disease/
95.	"multiple sclerosis".mp. [mp=title, abstract, heading word, drug trade name, original title, device manufacturer, drug manufacturer, device trade name, keyword, floating subheading word, candidate term word]
96.	exp Multiple Sclerosis/
97.	exp migraine/ or migraine.mp.
98.	meningitis.mp. or exp meningitis/
99.	exp encephalitis/ or encephalitis.mp.
100.	tetanus.mp. or exp tetanus/
101.	epilepsy.mp. or exp epilepsy/
102.	"brain cancer".mp. or exp brain metastasis/ or exp brain cancer/
103.	"brain tumo?r".mp. or exp brain tumor/
104.	76 or 77 or 78 or 79 or 80 or 81 or 82 or 83 or 84 or 85 or 86 or 87 or 88 or 89 or 90 or 91 or 92 or 93 or 94 or 95 or 96 or 97 or 98 or 99 or 100 or 101 or 102 or 103
105.	37 and 66 and 75 and 104
106.	limit 105 to (english language and yr="2008 -Current")
107.	limit 106 to (article or article in press or "review")

4. CINAHL via EBSCOhost

#	Query	Limiters/Expanders	Last Run Via
S1	(implement* OR effectiveness-implementation OR disseminat* OR diffus* OR utili* OR sustainab* OR uptake* OR facilitat* OR barrier* OR ((knowledge or research or technology) AND (translat* OR transform* OR exchange OR transfer OR integration OR utili?ation)) OR scalab* OR "Process evaluation" OR "process measure*" OR Feasib* OR adopt* OR adapt* OR usab* OR "lessons learned" OR implications OR experiences OR interoperab* OR fail* OR succes* OR acceptab* OR appropriate* OR fidelity OR adhere* OR complian* OR penetration OR cost* OR satisfaction OR hybrid OR pragmatic OR co-design OR codesign OR participatory* OR (MH "Cost Benefit Analysis") OR (MH "Action Research") OR (MH "Health Care Costs") OR (MH "Costs and Cost Analysis") OR (MH "Systems Implementation") OR (MH "Implementation Science") OR (MH "Program Implementation")) AND (telehealth OR tele-health OR telepractice OR tele-practice OR mHealth or m-Health OR “health telematics” OR tele-medicine OR computeri?ed OR internet-delivered OR internet-based OR online OR web-based OR “digital health” OR e-health OR ehealth OR “wireless health” OR e-therapy OR etherapy OR “healthcare technology” OR e-rehabilitation OR erehabilitation OR “User-Computer Interface” OR "digital therapy" OR “digital therapeutic*” OR e-mental OR emental OR “digital mental health” OR (MH "telehealth+")) AND (((behavio#ral OR behavio#r OR cognitive OR “cognitive behavio#ral” OR psychoeducation* OR psycho-education* OR psychosocial OR psycholog* OR dyad* OR group OR family OR caregiver OR “communication partner” OR communication) AND (therapy OR intervention OR medicine OR training OR treatment OR program OR rehabilitation OR coaching OR education or support)) OR (MH "Cognitive Therapy") OR (MH "Caregiver Emotional Health (Iowa NOC)") OR (MH "Mental Health Organizations") OR (MH "Community Mental Health Services") OR (MH "Rehabilitation, Cognitive") OR (MH "Mental Health Services") OR (MH "Cognitive Therapy (Iowa NIC)")OR (MH "Education+") OR "education" OR (MH "Communication+") OR "communication" OR (MM "Communication Skills Training")) AND (stroke OR “cerebrovascular accident” OR CVA OR (MM "Stroke Patients") OR (MH "Stroke")OR “traumatic brain injury” OR (MM "Head Injuries+") OR "head injury" OR “brain injury” OR TBI OR neurotrauma	Limiters - Published Date: 20080101-20201231; English Language; Peer Reviewed<br>Expanders - Apply equivalent subjects; Apply related words<br>Search modes - Boolean/Phrase	Interface - EBSCOhost Research Databases<br>Search Screen - Basic Search<br>Database - CINAHL

	OR (MH "Brain Injuries+") OR (MM "Right Hemisphere Injuries") OR (MM "International Brain Injury Association") OR ""brain injury"" OR (MM "Left Hemisphere Injuries") OR (MM "Aphasia+") OR "aphasia" OR "dysphasia" OR “Primary Progressive Aphasia” OR (MM "Dementia+") OR "dementia" OR (MM "Alzheimer's Disease") OR "alzheimer*" OR (MM "Parkinson Disease") OR "parkinson*" OR "motor neuron# disease" OR "mnd" OR (MM "Motor Neuron Diseases+") OR (MM "Multiple Sclerosis+") OR "multiple sclerosis" OR (MM "Migraine") OR "migraine" OR (MM "Meningitis+") OR "meningitis" OR(MM "Brain Neoplasms+") OR "brain cancer" OR "brain tumo#r" OR (MM "Meningitis+") OR "meningitis" OR(MM "Encephalitis+") OR "encephalitis" OR (MM "Tetanus") OR "tetanus"(MM "Epilepsy+") OR "epilepsy")		

5. PsycINFO via EBSCOhost

#	Query	Limiters/Expanders	Last Run Via
S9	S5 AND S6	Limiters - Publication Year: 2008-2020<br>Expanders - Apply related words; Apply equivalent subjects<br>Search modes - Boolean/Phrase	Interface - EBSCOhost Research Databases<br>Search Screen - Advanced Search<br>Database - APA PsycInfo
S8	S5 AND S6	Limiters - Publication Year: 2008-2020<br>Expanders - Apply related words; Apply equivalent subjects<br>Search modes - Boolean/Phrase	Interface - EBSCOhost Research Databases<br>Search Screen - Advanced Search<br>Database - APA PsycInfo
S7	S5 AND S6	Expanders - Apply related words; Apply equivalent subjects<br>Search modes - Boolean/Phrase	Interface - EBSCOhost Research Databases<br>Search Screen - Advanced Search<br>Database - APA PsycInfo
S6	S3 AND S4	Expanders - Apply related words; Apply equivalent subjects	Interface - EBSCOhost Research

		Search modes - Boolean/Phrase	Databases<br>Search Screen - Advanced Search<br>Database - APA PsycInfo
S5	S1 AND S2	Expanders - Apply related words; Apply equivalent subjects<br>Search modes - Boolean/Phrase	Interface - EBSCOhost Research Databases<br>Search Screen - Advanced Search<br>Database - APA PsycInfo
S4	stroke OR cerebrovascular accident OR CVA OR MM "Cerebrovascular Accidents" OR traumatic brain injury OR head injury OR brain injury OR TBI OR neurotrauma OR MM "Traumatic Brain Injury" OR MM "Brain Concussion" OR MM "Brain Injuries" OR MM "Aphasia" OR MM "Dysphasia" OR MM "Dysphasia" OR MM "Alexia" OR "aphasia" OR "primary progressive aphasia" OR "dysphasia" OR MM "Dementia" OR MM "AIDS Dementia Complex" OR MM "Dementia with Lewy Bodies" OR MM "Presenile Dementia" OR MM "Pseudodementia" OR MM "Semantic Dementia" OR MM "Senile Dementia" OR MM "Vascular Dementia" or "dementia" OR MM "Alzheimer's Disease" OR alzheimer* OR MM "Parkinson's Disease" OR MM "Parkinsonism" OR parkinson* OR "motor neuron# disease" OR "mnd" OR MM "Multiple Sclerosis" OR "multiple sclerosis" OR MM "Migraine Headache" or "migraine" OR MM "Meningitis" OR MM "Bacterial Meningitis" OR "meningitis" OR "Encephalitis" OR MM "Encephalitis" OR "tetanus" OR "epilepsy" or MM "Epilepsy" OR "brain tumo#r" OR "brain cancer" OR MM "Brain Neoplasms"	Expanders - Apply related words; Apply equivalent subjects<br>Search modes - Boolean/Phrase	Interface - EBSCOhost Research Databases<br>Search Screen - Advanced Search<br>Database - APA PsycInfo
S3	((behavio#ral OR behavio#r OR cognitive OR “cognitive behavio#ral” OR psychoeducation* OR psycho-education* OR psychosocial OR psycholog* OR dyad* OR group OR family OR caregiver OR “communication partner” OR communication) AND (therapy OR intervention OR medicine OR training OR treatment OR program OR rehabilitation OR coaching OR education OR support)) OR MM "Psychosocial	Expanders - Apply related words; Apply equivalent subjects	Interface - EBSCOhost Research Databases<br>Search Screen -

	Rehabilitation" OR MM "Psychosocial Readjustment" OR MM "Therapeutic Social Clubs" OR MM "Vocational Rehabilitation" OR DE "Cognitive Rehabilitation" OR MM "Behavior Therapy" OR MM "Aversion Therapy" OR MM "Conversion Therapy" OR MM "Dialectical Behavior Therapy" OR MM "Exposure Therapy" OR MM "Implosive Therapy" OR MM "Reciprocal Inhibition Therapy" OR MM "Response Cost" OR MM "Systematic Desensitization Therapy" OR MM "Caregiver Burden" OR MM "Caregivers" OR MM "Communication Skills Training"	Search modes - Boolean/Phrase	Advanced Search Database - APA PsycInfo
S2	telehealth OR tele-health OR telepractice OR tele-practice OR mHealth OR m-Health OR “health telematics” OR telehealth OR tele-medicine OR computeri?ed OR internet-delivered OR internet-based OR online OR web-based OR “digital health” OR e-health OR ehealth OR “wireless health” OR e-therapy OR etherapy OR “healthcare technology” OR e-rehabilitation OR erehabilitation OR “User-Computer Interface” OR "digital therapy" OR “digital therapeutic*” OR e-mental OR emental OR “digital mental health” OR MM "Telemedicine" OR MM "Teleconferencing" OR MM "Online Therapy" OR MM "Teleconsultation" OR MM "Telepsychiatry" OR MM "Telepsychology" OR MM "Telerehabilitation" OR MM "Online Therapy" OR MM "Telepsychology" OR MM "Telerehabilitation" OR MM "Digital Interventions"	Expanders - Apply related words; Apply equivalent subjects<br>Search modes - Boolean/Phrase	Interface - EBSCOhost Research Databases<br>Search Screen - Advanced Search<br>Database - APA PsycInfo
S1	implement* OR effectiveness-implementation OR disseminat* OR diffus* OR utili* OR sustainab* OR uptake* OR facilitat* OR barrier* OR ((knowledge or research or technology) AND (translat* OR transform* OR exchange OR transfer OR integration OR utili?ation)) OR scalab* OR "process evaluation" OR "Process measure*" OR Feasib* OR adopt* OR adapt* OR usab* OR "lessons learned" OR implications OR experiences OR interoperab* OR fail* OR succes* OR acceptab* OR appropriate* OR fidelity OR adhere* OR complian* OR penetration OR cost* OR satisfaction OR hybrid OR pragmatic OR co-design OR codesign OR participatory* OR MM "Knowledge Transfer" OR MM "Action Research" OR MM "Treatment Process and Outcome Measures" OR MM "Patient Reported Outcome Measures" OR MM "Health Care Delivery" OR MM "Clinical Practice" OR MM "Health Care Access" OR MM "Health Care Costs" OR MM "Health Care Reform" OR MM "Health Care Utilization" OR MM "Managed Care" OR MM "Quality of Care" OR MM "Quality of Services" OR MM "Health Care Utilization" OR MM "Utilization Reviews" OR MM "Stakeholder" OR MM "Innovation"	Expanders - Apply related words; Apply equivalent subjects<br>Search modes - Boolean/Phrase	Interface - EBSCOhost Research Databases<br>Search Screen - Advanced Search<br>Database - APA PsycInfo

6. SpeechBITE

For each of the below keywords:

**Keywords**	Implementation
	feasible
	Feasibility
	Adherence
	acceptable
	Acceptability
	Appropriate
	Appropriateness
	Fidelity
	"Process measure"
	"Process evaluation"
	Sustainability
	Satisfaction
	Utilisation
	utilization
	Usabillity
	Barriers
	Facilitators
	"lessons learned"
	effectiveness-implementation
	hybrid
	dissemination
	penetration
	co-design
	codesign
	participatory
	pragmatic
	diffusion
	cost
	adoption

	uptake
	scalable
	scalability
	inter-operability
	interoperability
	adapting
	adaptation
	implications
	failure
	success
	"knowledge transfer"
	"knowledge exchange"
	"research translation"
	integration

A search was run for each of two types of service delivery modes:

**Service delivery mode**	Computers and technology
	Distance

For each of the following populations:

Traumatic brain injury	Alzheimers and other dementias	Cancer	Degenerative disorders /diseases	Neurological conditions - other	Stroke/ CVA

With the following date limitations:

**Year**	2008-2020

7. NeuroBITE

Keywords	Implementation
	feasible
	Feasibility
	Adherence
	acceptable
	Acceptability
	Appropriate
	Appropriateness
	Fidelity
	Process measure
	Process evaluation
	sustainable
	Sustainability
	Satisfaction
	Utilization
	Utilisation
	Usabillity
	Barriers
	Facilitators
	"lessons learned"
	effectiveness-implementation
	hybrid
	dissemination
	penetration
	codesign
	co-design
	participatory
	pragmatic
	diffusion
	cost
	adoption
	uptake

	scalable
	scalability
	inter-operability
	interoperability
	adapting
	adaptation
	implications
	failure
	success
	knowledge transfer
	knowledge exchange
	research translation
	integration

**Year**	2008-2020

**Language**	English

**Target area**	Cognition / Mental	Language / Communication / Speech
	Behaviour / Emotion	Social Skills
	Other	Caregiver

**Intervention**	Carer training / Education	
	Cognitive	Cognitive / Neuropsychological Rehabilitation
		Communication / Language / Speech
	Equipment / Assistive devices	Computers and technology
	Psychological	CBT/ ACT / Behaviour therapy or modification
		Social skills
		Family support
		Counselling / psychotherapy
		Education / Psychoeducation / Bibliotherapy

**Method**	Group studies	RCTs
		Nonrandomised CTs
		Case series
	Single-case design	Experimental
		Non-experimental

**Service delivery mode**	Computers and technology
	Distance

**Neurological group**	Brain infections
	Brain tumours / neoplasms
	Drug and alcohol related brain injury / disorders
	Non specified brain impairment
	Epilepsy/ seizures / convulsions
	Hypoxia / anoxia
	MS
	Neurotoxicity
	Stroke / CVA
	TBI
