# Supplementary material for: Implementation of Web-Based Psychosocial Interventions for Adults With Acquired Brain Injury and Their Caregivers: Systematic Review
Source: J Med Internet Res. 2022 Jul 26;24(7):e38100. doi: 10.2196/38100 (PMC9328122; doi:10.2196/38100)
Supplement: Multimedia Appendix 2 [file jmir_v24i7e38100_app2.pdf]

Systematic Review NASSS Data Extraction

Please check the below responses against the article.

Thank you!

1 of 5 - Bibliographic details

Title ______________________________

Abstract ______________________________

First author surname ______________________________

Publication Year ______________________________

DOI URL ______________________________

2 of 5 - Summary

What is the target condition?

- ☐ TBI
- ☐ Stroke
- ☐ Brain Tumour
- ☐ Aphasia origin unspecified

Extent of user personalisation based on user characteristics or preference

- ◯ Completely individualised
- ◯ Partly individualised
- ◯ Not individualised
- ◯ No information

How does individualisation occur?

- ☐ Via clinician
- ☐ Via AI / Automation (e.g. embedded logic)
- ☐ Via user preference

Describe how individualisation is done: ______________________________

Intervention structure

- ◯ Tunnelling (guiding a user along a path)
- ◯ Free surfing
- ◯ Other (please describe)

____

What is the psychosocial intervention?	◯ Cognitive Behaviour Therapy (CBT)<br>◯ Communication Partner Training (CPT)<br>◯ Brain Training (Gamification)<br>◯ Education<br>◯ Other
Please specify what psychosocial intervention	__________________________________
What mode has been used to present the content?	☐ Images<br>☐ Audio<br>☐ Video<br>☐ Text<br>☐ Interactive games<br>☐ Telehealth videoconferencing<br>☐ Virtual Reality<br>☐ Instant Messaging<br>☐ Forum/ Message Board<br>☐ Email<br>☐ Productivity tool (e.g. calendar, note-taking application)<br>☐ No information
Who can participants interact with?	☐ Other PWABI<br>☐ Other caregivers<br>☐ Clinician<br>☐ AI<br>☐ No interaction
What platform is used?	◯ Desktop web-based<br>◯ Smartphone mobile application<br>◯ Tablet mobile application<br>◯ Both tablet and smartphone mobile application<br>◯ Desktop and mobile application<br>◯ Desktop and tablet<br>◯ No information
Does the intervention include:	◯ Person with brain injury only<br>◯ Both informal caregivers and the person with brain injury<br>◯ Informal caregivers only<br>◯ Formal caregivers only<br>◯ Both formal caregivers and the person with brain injury
Intervention setting	☐ Home<br>☐ School<br>☐ Community Health<br>☐ Hospital<br>☐ Other
What other setting?	__________________________________
How automatised is the intervention?	◯ Completely clinician-led i.e. telehealth only<br>◯ Partly automatised, partly telehealth<br>◯ Completely automatised / self-guided

3 of 5 - Evidence Appraisal

MMATS1 Are there clear research questions?

◯ Yes
◯ No
◯ Can't tell

MMATS1 What are the research questions?

____

MMATS2 Do the collected data allow the research questions to be addressed?

◯ Yes
◯ No
◯ Can't tell

What was the study design?

◯ Qualitative - exploring and understanding the meaning individuals or groups ascribe to a social or human problem
◯ Quantitative RCT - A clinical study in which individual participants are allocated to intervention or control groups by randomization (intervention assigned by researchers
◯ Quantitative non-randomised - any quantitative studies estimating the effectiveness of an intervention or studying other exposures that do not use randomization to allocate units to comparison groups.
◯ Quantitative descriptive - "concerned with and designed only to describe the existing distribution of variables without much regard to causal relationships or other hypotheses". They are used for monitoring the population, planning, and generating hypothesis.
◯ Mixed methods - (a) at least one QUAL method and one QUAN method are combined; (b) each method is used rigorously in accordance to the generally accepted criteria in the area (or tradition) of research invoked; and (c) the combination of the methods is carried out at the minimum through a MM design (defined a priori, or emerging) and the integration of the QUAL and QUAN phases, results, and data.
◯ Other

---

e.g.

Ethnography The aim of the study is to describe and interpret the shared cultural behaviour of a group of individuals.

Phenomenology The study focuses on the subjective experiences and interpretations of a phenomenon encountered by individuals.

Narrative research The study analyzes life experiences of an individual or a group.

Grounded theory Generation of theory from data in the process of conducting research (data collection occurs first).

Case study In-depth exploration and/or explanation of issues intrinsic to a particular case. A case can be anything from a decision-making process, to a person, an organization, or a country.

Qualitative description There is no specific methodology, but a qualitative data collection and analysis, e.g., in-depth interviews or focus groups, and hybrid thematic analysis (inductive and deductive).

e.g.

Non-randomized controlled trials: The intervention is assigned by researchers, but there is no randomization, e.g., a pseudo-randomization. A non-random method of allocation is not reliable in producing alone similar groups.

Cohort study: Subsets of a defined population are assessed as exposed, not exposed, or exposed at different degrees to factors of interest. Participants are followed over time to determine if an outcome occurs (prospective longitudinal).

Case-control study: Cases e.g., patients, associated with a certain outcome are selected, alongside a corresponding group of controls. Data is collected on whether cases and controls were exposed to the factor under study (retrospective).

Cross-sectional analytic study: At one particular time, the relationship between health-related characteristics (outcome) and other factors (intervention/exposure) is examined. E.g., the frequency of outcomes is compared in different population subgroups according to the presence/absence (or level) of the intervention/exposure.

Example single-group studies:

Incidence or prevalence study without comparison group: In a defined population at one particular time, what is happening in a population, e.g., frequencies of factors (importance of problems), is described (portrayed).

Survey: "Research method by which information is gathered by asking people questions on a specific topic and the data collection procedure is standardized and well defined."

Case series: A collection of individuals with similar characteristics are used to describe an outcome.

Case report: An individual or a group with a unique/unusual outcome is described in detail.)

E.g.

Convergent design: The QUAL and QUAN components are usually (but not necessarily) concomitant. The purpose is to examine the same phenomenon by interpreting QUAL and QUAN results (bringing data analysis together at the interpretation stage), or by integrating QUAL and QUAN datasets (e.g., data on same cases), or by transforming data (e.g., quantization of qualitative data).

Sequential explanatory design: Results of the phase 1 - QUAN component inform the phase 2 - QUAL component. The purpose is to explain QUAN results using QUAL findings. E.g., the QUAN results guide the selection of QUAL data sources and data collection, and the QUAL findings contribute to the interpretation of QUAN results.

Sequential exploratory design: Results of the phase 1 - QUAL component inform the phase 2 - QUAN component. The purpose is to explore, develop and test an instrument (or taxonomy), or a conceptual framework (or theoretical model). E.g., the QUAL findings inform the QUAN data collection, and the QUAN results allow a statistical generalization of the QUAL findings.

Specify study design. __________________________________

How many "Yes" values in MMAT?

- ◯ 0%
- ◯ 20%
- ◯ 40%
- ◯ 60%
- ◯ 80%
- ◯ 100%

MMAT1.1 Is the qualitative approach appropriate to answer the research question?

◯ Yes
◯ No
◯ Can't tell
(The qualitative approach used in a study should be appropriate for the research question and problem. "Qualitative research is an approach for exploring and understanding the meaning individuals or groups ascribe to a social or human problem" (Creswell, 2013b, p. 3). Common qualitative research approaches include (this list if not exhaustive): Ethnography The aim of the study is to describe and interpret the shared cultural behaviour of a group of individuals. Phenomenology The study focuses on the subjective experiences and interpretations of a phenomenon encountered by individuals. Narrative research The study analyzes life experiences of an individual or a group. Grounded theory Generation of theory from data in the process of conducting research (data collection occurs first). Case study In-depth exploration and/or explanation of issues intrinsic to a particular case. A case can be anything from a decision-making process, to a person, an organization, or a country. Qualitative description There is no specific methodology, but a qualitative data collection and analysis, e.g., in-depth interviews or focus groups, and hybrid thematic analysis (inductive and deductive). Key references: Creswell (2013a); Sandelowski (2010); Schwandt (2015))

MMAT1.2 Are the qualitative data collection methods adequate to address the research question?

◯ Yes
◯ No
◯ Can't tell
(This criterion is related to data collection method, including data sources (e.g., archives, documents), used to address the research question. To judge this criterion, consider whether the method of data collection (e.g., in depth interviews and/or group interviews, and/or observations) and the form of the data (e.g., tape recording, video material, diary, photo, and/or field notes) are adequate. Also, clear justifications are needed when data collection methods are modified during the study.)

MMAT1.3 Are the findings adequately derived from the data?

◯ Yes
◯ No
◯ Can't tell
(This criterion is related to the data analysis used. Several data analysis methods have been developed and their use depends onthe research question and qualitative approach. For example, open, axial and selective coding is often associated with grounded theory, and within- and cross-case analysis is often seen in case study.)

MMAT 1.4 Is the interpretation of results sufficiently substantiated by data?

◯ Yes
◯ No
◯ Can't tell
(The interpretation of results should be supported by the data collected. For example, the quotes provided to justify the themes should be adequate.
)

MMAT 1.4 Is there coherence between qualitative data sources, collection, analysis and interpretation?

◯ Yes
◯ No
◯ Can't tell
(There should be clear links between data sources, collection, analysis and interpretation.)

MMAT 2.1 Is randomization appropriately performed?

◯ Yes
◯ No
◯ Can't tell
(In a randomized controlled trial, the allocation of a participant (or a data collection unit, e.g., a school) into the intervention or control group is based solely on chance. Researchers should describe how the randomization schedule was generated. A simple statement such as 'we randomly allocated' or 'using a randomized design' is insufficient to judge if randomization was appropriately performed. Also, assignment that is predictable such as using odd and even record numbers or dates is not appropriate. At minimum, a simple allocation (or unrestricted allocation) should be performed by following a predetermined plan/sequence. It is usually achieved by referring to a published list of random numbers, or to a list of random assignments generated by a computer. Also, restricted allocation can be performed such as blocked randomization (to ensure particular allocation ratios to the intervention groups), stratified randomization (randomization performed separately within strata), or minimization (to make small groups closely similar with respect to several characteristics). Another important characteristic to judge if randomization was appropriately performed is allocation concealment that protects assignment sequence until allocation. Researchers and participants should be unaware of the assignment sequence up to the point of allocation. Several strategies can be used to ensure allocation concealment such relying on a central randomization by a third party, or the use of sequentially numbered, opaque, sealed envelopes (Higgins et al., 2016).)

MMAT2.2 Are the groups comparable at baseline?

◯ Yes
◯ No
◯ Can't tell
(Baseline imbalance between groups suggests that there are problems with the randomization. Indicators from baseline imbalance include: "(1) unusually large differences between intervention group sizes; (2) a substantial excess in statistically significant differences in baseline characteristics than would be expected by chance alone; (3) imbalance in key prognostic factors (or baseline measures of outcome variables) that are unlikely to be due to chance; (4) excessive similarity in baseline characteristics that is not compatible with chance; (5) surprising absence of one or more key characteristics that would be expected to be reported" (Higgins et al., 2016, p. 10).)

MMAT2.3 Are there complete outcome data?

◯ Yes
◯ No
◯ Can't tell
(Almost all the participants contributed to almost all measures. There is no absolute and standard cut-off value for acceptable complete outcome data. Agree among your team what is considered complete outcome data in your field and apply this uniformly across all the included studies. For instance, in the literature, acceptable complete data value ranged from 80% (Thomas et al., 2004; Zaza et al., 2000) to 95% (Higgins et al., 2016). Similarly, different acceptable withdrawal/dropouts rates have been suggested: 5% (de Vet et al., 1997; MacLehose et al., 2000), 20% (Sindhu et al., 1997; Van Tulder et al., 2003) and 30% for a follow-up of more than one year (Viswanathan and Berkman, 2012).)

MMAT2.4 Are outcome assessors blinded to the intervention provided?

◯ Yes
◯ No
◯ Can't tell
(Outcome assessors should be unaware of who is receiving which interventions. The assessors can be the participants if using participant reported outcome (e.g., pain), the intervention provider (e.g., clinical exam), or other persons not involved in the intervention (Higgins et al., 2016).)

MMAT2.5 Did the participants adhere to the assigned intervention?

◯ Yes
◯ No
◯ Can't tell
(To judge this criterion, consider the proportion of participants who continued with their assigned intervention throughout follow-up. "Lack of adherence includes imperfect compliance, cessation of intervention, crossovers to the comparator intervention and switches to another active intervention." (Higgins et al., 2016, p. 25).)

MMAT3.1 Are the participants representative of the target population?

◯ Yes
◯ No
◯ Can't tell
(Indicators of representativeness include: clear description of the target population and of the sample (inclusion and exclusion criteria), reasons why certain eligible individuals chose not to participate, and any attempts to achieve a sample of participants that represents the target population.)

MMAT3.2 Are measurements appropriate regarding both the outcome and intervention (or exposure)?

◯ Yes
◯ No
◯ Can't tell
(Indicators of appropriate measurements include: the variables are clearly defined and accurately measured; the measurements are justified and appropriate for answering the research question; the measurements reflect what they are supposed to measure; validated and reliability tested measures of the intervention/exposure and outcome of interest are used, or variables are measured using 'gold standard'.)

MMAT3.3 Are there complete outcome data?

◯ Yes
◯ No
◯ Can't tell
(Almost all the participants contributed to almost all measures. There is no absolute and standard cut-off value for acceptable complete outcome data. Agree among your team what is considered complete outcome data in your field (and based on the targeted journal) and apply this uniformly across all the included studies. For example, in the literature, acceptable complete data value ranged from 80% (Thomas et al., 2004; Zaza et al., 2000) to 95% (Higgins et al., 2016). Similarly, different acceptable withdrawal/dropouts rates have been suggested: 5% (de Vet et al., 1997; MacLehose et al., 2000), 20% (Sindhu et al., 1997; Van Tulder et al., 2003) and 30% for follow-up of more than one year (Viswanathan and Berkman, 2012).)

MMAT3.4 Are the confounders accounted for in the design and analysis?

◯ Yes
◯ No
◯ Can't tell
(Confounders are factors that predict both the outcome of interest and the intervention received/exposure at baseline. They can distort the interpretation of findings and need to be considered in the design and analysis of a non-randomized study. Confounding bias is low if there is no confounding expected, or appropriate methods to control for confounders are used (such as stratification, regression, matching, standardization, and inverse probability weighting).)

MMAT3.5 During the study period, is the intervention administered (or exposure occurred) as intended?

◯ Yes
◯ No
◯ Can't tell

(For intervention studies, consider whether the participants were treated in a way that is consistent with the planned intervention. Since the intervention is assigned by researchers, consider whether there was a presence of contamination (e.g., the control group may be indirectly exposed to the intervention) or whether unplanned co-interventions were present in one group (Sterne et al., 2016). For observational studies, consider whether changes occurred in the exposure status among the participants. If yes, check if these changes are likely to influence the outcome of interest, were adjusted for, or whether unplanned co-exposures were present in one group (Morgan et al., 2017).)

Is the sampling strategy relevant to address the research question?

◯ Yes
◯ No
◯ Can't tell

(Sampling strategy refers to the way the sample was selected. There are two main categories of sampling strategies: probability sampling (involve random selection) and non-probability sampling. Depending on the research question, probability sampling might be preferable. Non-probability sampling does not provide equal chance of being selected. To judge this criterion, consider whether the source of sample is relevant to the target population; a clear justification of the sample frame used is provided; or the sampling procedure is adequate.)

Is the sample representative of the target population?

◯ Yes
◯ No
◯ Can't tell

(There should be a match between respondents and the target population. Indicators of representativeness include: clear description of the target population and of the sample (such as respective sizes and inclusion and exclusion criteria), reasons why certain eligible individuals chose not to participate, and any attempts to achieve a sample of participants that represents the target population)

Are the measurements appropriate?

◯ Yes
◯ No
◯ Can't tell

(Indicators of appropriate measurements include: the variables are clearly defined and accurately measured, the measurements are justified and appropriate for answering the research question; the measurements reflect what they are supposed to measure; validated and reliability tested measures of the outcome of interest are used, variables are measured using 'gold standard', or questionnaires are pre-tested prior to data collection.)

Is the risk of nonresponse bias low?	◯ Yes<br>◯ No<br>◯ Can't tell<br>(Nonresponse bias consists of "an error of nonobservation reflecting an unsuccessful attempt to obtain the desired information from an eligible unit." (Federal Committee on Statistical Methodology, 2001, p. 6). To judge this criterion, consider whether the respondents and non-respondents are different on the variable of interest. This information might not always be reported in a paper. Some indicators of low nonresponse bias can be considered such as a low nonresponse rate, reasons for nonresponse (e.g., noncontacts vs. refusals), and statistical compensation for nonresponse (e.g., imputation). The nonresponse bias is might not be pertinent for case series and case report. This criterion could be adapted. For instance, complete data on the cases might be important to consider in these designs.)
Is the statistical analysis appropriate to answer the research question?	◯ Yes<br>◯ No<br>◯ Can't tell<br>(The statistical analyses used should be clearly stated and justified in order to judge if they are appropriate for the design and research question, and if any problems with data analysis limited the interpretation of the results.)
Is there an adequate rationale for using a mixed methods design to address the research question?	◯ Yes<br>◯ No<br>◯ Can't tell<br>(The reasons for conducting a mixed methods study should be clearly explained. Several reasons can be invoked such as to enhance or build upon qualitative findings with quantitative results and vice versa; to provide a comprehensive and complete understanding of a phenomenon or to develop and test instruments (Bryman, 2006).)
Are the different components of the study effectively integrated to answer the research question?	◯ Yes<br>◯ No<br>◯ Can't tell<br>(Integration is a core component of mixed methods research and is defined as the "explicit interrelating of the quantitative and qualitative component in a mixed methods study" (Plano Clark and Ivankova, 2015, p. 40). Look for information on how qualitative and quantitative phases, results, and data were integrated (Pluye et al., 2018). For instance, how data gathered by both research methods was brought together to form a complete picture (e.g., joint displays) and when integration occurred (e.g., during the data collection-analysis or/and during the interpretation of qualitative and quantitative results).)

Are the outputs of the integration of qualitative and quantitative components adequately interpreted?	◯ Yes<br>◯ No<br>◯ Can't tell<br>(This criterion is related to meta-inference, which is defined as the overall interpretations derived from integrating qualitative and quantitative findings (Teddlie and Tashakkori, 2009). Meta-inference occurs during the interpretation of the findings from the integration of the qualitative and quantitative components, and shows the added value of conducting a mixed methods study rather than having two separate studies.)
Are divergences and inconsistencies between quantitative and qualitative results adequately addressed?	◯ Yes<br>◯ No<br>◯ Can't tell<br>(When integrating the findings from the qualitative and quantitative components, divergences and inconsistencies (also called conflicts, contradictions, discordances, discrepancies, and dissonances) can be found. It is not sufficient to only report the divergences; they need to be explained. Different strategies to address the divergences have been suggested such as reconciliation, initiation, bracketing and exclusion (Pluye et al., 2009b). Rate this criterion 'Yes' if there is no divergence.)
Do the different components of the study adhere to the quality criteria of each tradition of the methods involved?	◯ Yes<br>◯ No<br>◯ Can't tell<br>(The quality of the qualitative and quantitative components should be individually appraised to ensure that no important threats to trustworthiness are present. To appraise 5.5, use criteria for the qualitative component (1.1 to 1.5), and the appropriate criteria for the quantitative component (2.1 to 2.5, or 3.1 to 3.5, or 4.1 to 4.5). The quality of both components should be high for the mixed methods study to be considered of good quality. The premise is that the overall quality of a mixed methods study cannot exceed the quality of its weakest component. For example, if the quantitative component is rated high quality and the qualitative component is rated low quality, the overall rating for this criterion will be of low quality.)
This study examined implementation experiences of:	☐ PWABI<br>☐ Caregivers<br>☐ Clinicians<br>☐ Administrative staff<br>☐ Other
What was the sample size of people with the condition receiving the digital intervention? n=	__________________________________<br>(0 if caregivers only)
What was the sample size of people with the condition in the comparison/control group? n=	__________________________________<br>(0 if no control/comparison)

What was the sample size of caregivers? n=	__________________ (0 if no caregivers in this study)
What was the sample size of caregivers in the control/comparison group? n=	__________________ (0 if no caregiver control/comparison in this study)
What was the sample size of clinicians under study delivering the intervention? n=	__________________ (0 if no clinicians in this study)
What was the sample size of clinicians in the control/comparison group under study? n=	__________________ (0 if no clinician control/comparison in this study)
What was the sample size of admin staff under study delivering the intervention? n=	__________________ (0 if no admin staff in this study)
What was the sample size of admin staff in the control/comparison group under study? n=	__________________ (0 if no admin staff control/comparison in this study)
What was the sample size of others under study receiving the intervention? n=	__________________ (0 if no others in this study)
What was the sample size of others controls under study receiving the intervention? n=	__________________ (0 if no other controls in this study)
What was the implementation study design?	◯ Hybrid Type 1: Primary Aim: Determine effectiveness of an intervention, Secondary Aim: Better understand context for implementation<br>◯ Hybrid Type 2: Primary Aim: Determine effectiveness of an intervention, Co-Primary or Secondary Aim: Determine feasibility and/or (potential) impact of an implementation strategy<br>◯ Hybrid Type 3: Primary Aim: Determine impact of an implementation strategy, Secondary Aim: Assess clinical outcomes associated with implementation<br>◯ Qualitative<br>◯ Other

Which implementation outcome measures were included?	☐ Feasibility i.e., the extent to which it can be successfully deployed in a given setting<br>☐ Usability<br>☐ Acceptability i.e., how palatable or agreeable it is from the perspective of stakeholders<br>☐ Appropriateness i.e., the perceived fit of for a given setting, clinician, or patient<br>☐ Adherence/ Fidelity i.e., the degree to which it is implemented as it was intended<br>☐ Satisfaction<br>☐ Cost-effectiveness<br>☐ Other<br>(Select all that apply)
Feasibility measures	__________________________
Feasibility findings	__________________________
Usability measures	__________________________
Usability findings	__________________________
Acceptability measures	__________________________
Acceptability findings	__________________________
Appropriateness measures	__________________________
Appropriateness findings	__________________________
Fidelity/Adherence measures	__________________________
Fidelity/Adherence findings	__________________________
Satisfaction_measures	__________________________

Satisfaction_findings	__________________________________
Cost-effectiveness measures	__________________________________
Cost-effectiveness findings	__________________________________
What other implementation outcomes?	__________________________________
What other implementation results?	__________________________________
What was the scope of implementation?	◯ Single site<br>◯ Multiple sites<br>◯ State<br>◯ National<br>◯ International
Did the authors use an implementation framework or theory?	◯ Yes<br>◯ No
Please specify the framework/theory	__________________________________
How was the implementation theory/framework used?	☐ Inform intervention development<br>☐ Inform study design<br>☐ Inform outcome measures<br>☐ Data analysis/interpretation<br>☐ Other
Please specify how the theory/framework was used	__________________________________
Were people with brain injury, caregivers or clinicians involved in the intervention's design or evaluation?	☐ people with brain injury<br>☐ caregivers<br>☐ clinicians<br>☐ no<br>☐ no information
People with brain injury, caregivers or clinicians were involved in:	☐ Co-design of the intervention<br>☐ Co-design of the research<br>☐ Evaluation of the intervention<br>☐ Other
Please describe the method of stakeholder involvement	__________________________________

Were the implementation outcomes achieved?

- ◯ Yes
- ◯ No
- ◯ Can't tell

Summary of implementation outcome.

____

5 of 5 - NASSS framework and Bewick Taxonomy

Domain 1a The nature of the condition is:

- ◯ Simple: Well-characterised, well-understood, predictable
- ◯ Complicated: Not fully characterised, understood, or predictable
- ◯ Complex: Poorly characterised, poorly understood, unpredictable, or high risk
- ◯ No information

Domain 1a What is the nature of the condition?

____
(e.g. eligibility/inclusion criteria)

Domain 1b The relevant sociocultural factors and comorbidities are:

- ◯ Simple: Unlikely to affect care significantly
- ◯ Complicated: Must be factored into care plan and service model
- ◯ Complex: Pose significant challenges to care planning and service provision
- ◯ No information

Domain 1b What are the relevant sociocultural factors and comorbidities?

____
(e.g. exclusion criteria)

Domain 2a The material and technical features of the technology were:

- ◯ Simple: Off-the-shelf or already installed, freestanding, dependable
- ◯ Complicated: Not yet developed or fully interoperable; not 100% dependable
- ◯ Complex: Requires close embedding in complex technical systems; significant dependability issues
- ◯ No information

Domain 2a What were the material and technical features of the technology?

____
(Material and technical features of the technology)

Domain 2b The kind of knowledge generated or made visible by the technology was:

- ◯ Simple: Directly and transparently measures [changes in] the condition
- ◯ Complicated: Partially and indirectly measures [changes in] the condition
- ◯ Complex: Link between data generated and [changes in] the condition is currently unpredictable or contested
- ◯ No information

(This includes not only the accuracy of the data but also the extent to which those data are accepted, trusted, and considered sufficient for decision making. Engaging with the data generated by patient-facing technologies may inform, educate, and empower patients and lay caregivers.)

Domain 2b What kind of knowledge was generated or made visible by the technology?

____

(This includes not only the accuracy of the data but also the extent to which those data are accepted, trusted, and considered sufficient for decision making. Engaging with the data generated by patient-facing technologies may inform, educate, and empower patients and lay caregivers.)

Mode of data entry

- ☐ By self
- ☐ By clinician
- ☐ Automatic
- ☐ None

Domain 2c The knowledge and support needed to use the technology was:

- ◯ Simple: None or a simple set of instructions
- ◯ Complicated: Detailed instruction and training needed, perhaps with ongoing helpdesk support
- ◯ Complex: Effective use of technology requires advanced training and/or support to adjust to new identity or organisational role
- ◯ No information

(Some technologies are much easier to operate than others; some require frequent troubleshooting; and some assume a different organisational role-or even an altered professional identity-for the user. Some patient-facing technologies require no knowledge from the patient; others require clinical knowledge, technical knowledge, and the ability to make judgments about (for example) what counts as urgent.)

Domain 2c What knowledge and support was needed to use the technology?

____

(Some technologies are much easier to operate than others; some require frequent troubleshooting; and some assume a different organisational role-or even an altered professional identity-for the user. Some patient-facing technologies require no knowledge from the patient; others require clinical knowledge, technical knowledge, and the ability to make judgments about (for example) what counts as urgent.)

Domain 2d The technology supply model was:

- ◯ Simple: Generic, "plug and play," or customisable, off-the-shelf solutions requiring minimal customisation; easily substitutable if supplier withdraws
- ◯ Complicated: customisable, off-the-shelf solutions requiring significant customisation or bespoke solutions; substitution difficult if supplier withdraws
- ◯ Complex: Solutions requiring significant organisational reconfiguration or medium- to large scale bespoke solutions, highly vulnerable to supplier withdrawal
- ◯ No information

(How the technology was procured, the nature of the client-supplier relationship, and the level of potential substitutability via the marketplace.)

Domain 2d What was the technology supply model?

____

(How the technology was procured, the nature of the client-supplier relationship, and the level of potential substitutability via the marketplace.)

Domain 2e Who owned the intellectual property (IP) generated by the technology?

____

Domain 3a The developer's business case for the technology (supply-side value to the developer) was:

- ◯ Simple: Clear business case with strong chance of return on investment
- ◯ Complicated: Business case underdeveloped; potential risk to investors
- ◯ Complex: Business case implausible; significant risk to investors
- ◯ No information

(Addresses upstream value, which follows the supply-side logic of financial markets and investment decisions (and hence depends on preliminary tests of efficacy and safety, and evidence of good business practice).)

Domain 3a What was the developer's business case for the technology (supply-side value to the developer)?

____

(Addresses upstream value, which follows the supply-side logic of financial markets and investment decisions (and hence depends on preliminary tests of efficacy and safety, and evidence of good business practice).)

Domain 3b The desirability, efficacy, safety, and cost effectiveness (demand-side value to the patient) was:

- ◯ Simple: Technology is desirable for patients, effective, safe, and cost effective
- ◯ Complicated: Technology's desirability, efficacy, safety, or cost effectiveness is unknown or contested
- ◯ Complex: Significant possibility that technology is undesirable, unsafe, ineffective, or unaffordable
- ◯ No information

(Addresses downstream value, which follows the demand-side logic of health technology appraisal, reimbursement, and procurement (ie, relates to evidence of benefit to patients and real-world affordability).)

Domain 3b What was its desirability, efficacy, safety, and cost effectiveness (demand-side value to the patient)?

____

(Addresses downstream value, which follows the demand-side logic of health technology appraisal, reimbursement, and procurement (ie, relates to evidence of benefit to patients and real-world affordability).)

Buyer/ Availability

- ☐ Individual patients/families
- ☐ Individual clinicians
- ☐ Individual organisation/service
- ☐ Governments
- ☐ No information

Cost structure

- ☐ Free
- ☐ Subscription payment
- ☐ Upfront payment
- ☐ Other
- ☐ No information

Please describe the cost structure

____

Domain 4a The assumptions and requirements made of clinicians/implied changes in staff roles, practices and identities were:

- ◯ Simple: None
- ◯ Complicated: Existing staff must learn new skills and/or new staff be appointed
- ◯ Complex: Threat to professional identity, values, or scope of practice; risk of job loss
- ◯ No information

Domain 4a What assumptions and requirements were made of clinicians i.e. What changes in staff roles, practices and identities were implied?

____

Domain 4b Expectations of the patient-and whether this was achievable by, and acceptable to them were:

- ◯ Simple: Nothing
- ◯ Complicated: Routine tasks, eg, log on, enter data, converse
- ◯ Complex: Complex tasks, eg, initiate changes in therapy, make judgments, organise
- ◯ No information

((passive vs active input))

Domain 4b What assumptions and requirements were made of clients i.e. What was expected of the patient (and/or immediate carer) - and was this achievable by and acceptable to them?

____
((passive vs active input))

Amount of time per session/overall

____

Domain 4c Assumptions and requirements made about the extended network of lay carers are:

- ◯ Simple: None
- ◯ Complicated: Assumes a caregiver will be available when needed
- ◯ Complex: Assumes a network of caregivers with ability to coordinate their input
- ◯ No information

((availability, type of input))

Domain 4c What assumptions and requirements were made of caregivers i.e. What was assumed about the extended network of lay carers?

____
((availability, type of input))

Domain 5a The organisational capacity to innovate was:

- ◯ Simple: Well-led organization with slack resources and good managerial relations; risk taking encouraged
- ◯ Complicated: Limited slack resources; suboptimal leadership and managerial relations; risk taking not encouraged
- ◯ Complex: Severe resource pressures (eg, frozen posts); weak leadership and managerial relations; risk taking may be punished
- ◯ No information

Domain 5a What organisational capacity was there to innovate?

____

Domain 5b The readiness of the organisation for this technology-supported change was:

- ◯ Simple: High tension for change, good innovation-system fit, widespread support
- ◯ Complicated: Little tension for change; moderate innovation-system fit; some powerful opponents
- ◯ Complex: No tension for change; poor innovation-system fit; many opponents, some with wrecking power
- ◯ No information

Domain 5b How ready was the organisation for this technology-supported change?

____

Domain 5a The adoption and funding decision was:

- ◯ Simple: Single organisation with sufficient resources; anticipated cost savings; no new infrastructure or recurrent costs required
- ◯ Complicated: Multiple organisations with partnership relationship; cost-benefit balance favorable or neutral; new infrastructure (eg, staff roles, training, kit) can mostly be found from repurposing
- ◯ Complex: Multiple organisations with no formal links and/or conflicting agendas; funding depends on cost savings across system; costs and benefits unclear; new infrastructure conflicts with existing; significant budget implications
- ◯ No information

Domain 5c How easy was the adoption and funding decision?

____

Domain 5c The changes needed in team interactions and routines were:

- ◯ Simple: No new team routines or care pathways needed
- ◯ Complicated: New team routines or care pathways that align readily with established ones
- ◯ Complex: New team routines or care pathways that conflict with established ones
- ◯ No information

(Work needed to plan, implement and monitor change.)

Domain 5d What changes were needed in team interactions and routines?

____

(Work needed to plan, implement and monitor change.)

Domain 5e The work involved in implementation was:

- ◯ Simple: Established shared vision; few simple tasks, uncontested and easily monitored
- ◯ Complicated: Some work needed to build shared vision, engage staff, enact new practices, and monitor impact
- ◯ Complex: Significant work needed to build shared vision, engage staff, enact new practices, and monitor impact
- ◯ No information

Domain 5e What work was involved in implementation and who did it?

____

Country

- ◯ Afghanistan
- ◯ Albania
- ◯ Algeria
- ◯ Andorra
- ◯ Angola
- ◯ Antigua and Barbuda
- ◯ Argentina
- ◯ Armenia
- ◯ Australia
- ◯ Austria
- ◯ Azerbaijan
- ◯ Bahamas
- ◯ Bahrain
- ◯ Bangladesh
- ◯ Barbados
- ◯ Belarus
- ◯ Belgium
- ◯ Belize
- ◯ Benin
- ◯ Bhutan
- ◯ Bolivia
- ◯ Bosnia and Herzegovina
- ◯ Botswana
- ◯ Brazil
- ◯ Brunei Darussalam
- ◯ Bulgaria
- ◯ Burkina Faso
- ◯ Burundi
- ◯ Cambodia
- ◯ Cameroon
- ◯ Canada
- ◯ Cape Verde
- ◯ Central African Republic
- ◯ Chad
- ◯ Chile
- ◯ China
- ◯ Colombia
- ◯ Comoros
- ◯ Republic of the...
- ◯ Costa Rica
- ◯ Côte d'Ivoire
- ◯ Croatia
- ◯ Cuba
- ◯ Cyprus
- ◯ Czech Republic
- ◯ Democratic Republic of the Congo
- ◯ Denmark
- ◯ Djibouti
- ◯ Dominica
- ◯ Dominican Republic
- ◯ Ecuador
- ◯ Egypt
- ◯ El Salvador
- ◯ Equatorial Guinea
- ◯ Eritrea
- ◯ Estonia
- ◯ Ethiopia
- ◯ Fiji
- ◯ Finland
- ◯ France
- ◯ Gabon
- ◯ Gambia
- ◯ Georgia
- ◯ Germany
- ◯ Ghana
- ◯ Greece
- ◯ Grenada
- ◯ Guatemala
- ◯ Guinea

◯ Guinea-Bissau
◯ Guyana
◯ Haiti
◯ Honduras
◯ Hong Kong (S.A.R.)
◯ Hungary
◯ Iceland
◯ India
◯ Indonesia
◯ Iran
◯ Iraq
◯ Ireland
◯ Israel
◯ Italy
◯ Jamaica
◯ Japan
◯ Jordan
◯ Kazakhstan
◯ Kenya
◯ Kiribati
◯ Kuwait
◯ Kyrgyzstan
◯ Lao People's Democratic Republic
◯ Latvia
◯ Lebanon
◯ Lesotho
◯ Liberia
◯ Libyan Arab Jamahiriya
◯ Liechtenstein
◯ Lithuania
◯ Luxembourg
◯ Madagascar
◯ Malawi
◯ Malaysia
◯ Maldives
◯ Mali
◯ Malta
◯ Marshall Islands
◯ Mauritania
◯ Mauritius
◯ Mexico
◯ Federated States of...
◯ Monaco
◯ Mongolia
◯ Montenegro
◯ Morocco
◯ Mozambique
◯ Myanmar
◯ Namibia
◯ Nauru
◯ Nepal
◯ Netherlands
◯ New Zealand
◯ Nicaragua
◯ Niger
◯ Nigeria
◯ North Korea
◯ Norway
◯ Oman
◯ Pakistan
◯ Palau
◯ Panama
◯ Papua New Guinea
◯ Paraguay
◯ Peru
◯ Philippines
◯ Poland
◯ Portugal
◯ Qatar
◯ Republic of Moldova
◯ Romania

◯ Russian Federation
◯ Rwanda
◯ Saint Kitts and Nevis
◯ Saint Lucia
◯ Saint Vincent and the Grenadines
◯ Samoa
◯ San Marino
◯ Sao Tome and Principe
◯ Saudi Arabia
◯ Senegal
◯ Serbia
◯ Seychelles
◯ Sierra Leone
◯ Singapore
◯ Slovakia
◯ Slovenia
◯ Solomon Islands
◯ Somalia
◯ South Africa
◯ South Korea
◯ Spain
◯ Sri Lanka
◯ Sudan
◯ Suriname
◯ Swaziland
◯ Sweden
◯ Switzerland
◯ Syrian Arab Republic
◯ Tajikistan
◯ Thailand
◯ The former Yugoslav Republic of Macedonia
◯ Timor-Leste
◯ Togo
◯ Tonga
◯ Trinidad and Tobago
◯ Tunisia
◯ Turkey
◯ Turkmenistan
◯ Tuvalu
◯ Uganda
◯ Ukraine
◯ United Arab Emirates
◯ United Kingdom of Great Britain and Northern Ireland
◯ United Republic of Tanzania
◯ United States of America
◯ Uruguay
◯ Uzbekistan
◯ Vanuatu
◯ Bolivarian Republic of...
◯ Viet Nam
◯ Yemen
◯ Zambia
◯ Zimbabwe

(Country of authors)

Domain 6

What was the political context of implementation?

____

Domain 6b The regulatory context of implementation was:

- ◯ Simple: Financial and regulatory requirements already in place nationally
- ◯ Complicated: Financial and regulatory requirements being negotiated nationally
- ◯ Complex: Financial and regulatory requirements raise tricky legal or other challenges
- ◯ No information:

Domain 6b What was the regulatory context of implementation?

____

Domain 6c The professional context of implementation i.e. the position of professional bodies was:

- ◯ Simple: professional bodies supportive
- ◯ Complicated: professional stakeholders not yet committed
- ◯ Complex: professional bodies unsupportive or opposed
- ◯ No information

Domain 6c What was the professional context of implementation i.e. what was the position of professional bodies?

____

Domain 6d The sociocultural context of implementation i.e. (public perception, interest, expectation) was:

- ◯ Simple: civil society supportive
- ◯ Complicated: lay stakeholders not yet committed
- ◯ Complex: lay stakeholders unsupportive or opposed
- ◯ No information

Domain 6d What was the sociocultural context of implementation i.e. (public perception, interest, expectation)?

____

Domain 6e What was the interorganisational context of implementation i.e. What was the nature and extent of inter-organisational networking?

____

Domain 7a The scope for adapting and co-evolving the technology and the service over time was:

- ◯ Simple: Strong scope for adapting and embedding the technology as local need or context changes
- ◯ Complicated: Potential for adapting and coevolving the technology and service is limited or uncertain
- ◯ Complex: Significant barriers to further adaptation and/or coevolution of the technology or service
- ◯ No information

Domain 7a How much scope was there for adapting and co-evolving the technology and the service over time?

____

Domain 7b The resilience of the organisation to handling critical events and adapting to unforeseen eventualities was:

- ◯ Simple: Sense making, collective reflection, and adaptive action are ongoing and encouraged
- ◯ Complicated: Sense making, collective reflection, and adaptive action are difficult and viewed as low priority
- ◯ Complex: Sense making, collective reflection, and adaptive action are discouraged in a rigid, inflexible implementation model
- ◯ No information

Domain 7b How resilient was the organisation to handling critical events and adapting to unforeseen eventualities?

____
